# Supplementary material for: Protocol for a multicentre, parallel-group, open-label randomised controlled trial comparing ferric carboxymaltose with the standard of care in anaemic Malawian pregnant women: the REVAMP trial
Source: BMJ Open. 2021 Nov 23;11(11):e053288. doi: 10.1136/bmjopen-2021-053288 (PMC8611444; doi:10.1136/bmjopen-2021-053288)
Supplement: Supplementary data [file bmjopen-2021-053288supp001.pdf]

Supplementary Table 1: Primary, secondary and exploratory outcomes included in the trial registration for mothers and neonates.

	Protocol activity	Outcome type	Outcome	Timepoint
Mother	Laboratory procedures	Primary/secondary	Anaemia (Hb<11g/dL) as measured on venous blood via automated analyser	4 weeks post-intervention (for both IV iron and commencement of oral iron), week 36, at delivery, 4 weeks postpartum
		Secondary	Haemoglobin as measured on venous blood via automated analyser	4 weeks post-intervention (for both IV iron and commencement of oral iron), week 36, at delivery, 4 weeks postpartum
		Secondary	Ferritin as measured by serum ferritin	4 weeks post-intervention (for both IV iron and commencement of oral iron), week 36, at delivery, 4 weeks postpartum
		Exploratory	Iron deficiency by sTfR/Ferritin index assay	4 weeks post-intervention (for both IV iron and commencement of oral iron), week 36, at delivery, 4 weeks postpartum
		Secondary	Iron deficiency (ferritin < 15μg/L)	4 weeks post-intervention (for both IV iron and commencement of oral iron), week 36, at delivery, 4 weeks postpartum
		Exploratory	Incidence of placental malaria at delivery based on placental histologic examination	Delivery
		Exploratory	Incidence of peripheral parasitaemia by 36 weeks of gestation based on blood film microscopy	Randomisation to 36 weeks' gestation
		Exploratory	Prevalence of malaria parasitaemia based on blood film microscopy at each scheduled visit	4 weeks post-intervention (for both IV iron and commencement of oral iron), 36 weeks, at delivery, 4 weeks postpartum
		Safety	Hypophosphatemia based on biochemical measurement of serum Phosphate.	4 weeks post-intervention (for both Iv iron and commencement of oral iron), 36 weeks

		Safety	Inflammation (elevated C-reactive protein by serum assay)	4 weeks post-intervention (for both Iv iron and commencement of oral iron), 36 weeks' gestation
		Exploratory	Health systems costs of providing the treatments and follow-up for the intervention and comparator based on measurement of resource use and costing of relevant resources, with direct measurement of health care resource utilisation	Each planned visit that coincides with a pregnancy visit (baseline (second trimester), week 36, delivery), unplanned visits (e.g., during any episode of infection requiring management)
	Household economics	Exploratory	Direct and indirect patient costs including patient out-of-pocket costs for both health care and other costs, e.g., transport/ food, and lost income for receiving the intervention and the comparator	Each planned visit that coincides with a pregnancy visit (baseline (second trimester), week 36, delivery), unplanned visits (e.g., during any episode of infection requiring management)
		Exploratory	Fatigue measured by the Piper Fatigue Scale	4 weeks post-intervention (for both IV iron and commencement of oral iron), week 36, 4 weeks postpartum
	Maternal cognition	Exploratory	Cognitive function using digit span forward and backward test, and mental rotation tests	4 weeks post-intervention (for both IV iron and commencement of oral iron), 4 weeks postpartum
		Safety	Severe anaemia requiring blood transfusion as defined by clinical notes	From randomisation (receipt of oral or intravenous iron depending on allocation) to 4 weeks postpartum
	Adverse events	Safety	Severe medical events: shock (systolic blood pressure <90mmHg), need for blood transfusion, ICU admission, or mortality: individually and as a composite outcome, based on direct clinical observation by study staff	From randomisation (receipt of oral or intravenous iron depending on allocation) to 4 weeks postpartum

		Safety	Adverse events, as recorded by direct questioning of participants during administration visit. Such adverse events may include flushing, rash, allergic reactions, headache etc	Time of administration of the intervention
		Safety	Incidence of all-cause sick visits to the clinic based on visits recorded by study staff at the study clinic	Randomisation to delivery
		Exploratory	Incidence of diarrhoea sick visits to the clinic based on visits recorded by study staff at the study clinic	Randomisation to delivery
		Safety	Incidence of clinical malaria-specific sick visits to the clinic based on visits recorded by study staff at the study clinic	Randomisation to delivery
		Safety	Haemorrhage - antepartum or postpartum haemorrhage diagnosed by study clinical staff	Randomisation to 4 weeks postpartum
		Safety	Mortality	Randomisation to 1-month postpartum
		Exploratory	Shock defined by systolic blood pressure <90mmHg, as observed by study staff	Randomisation to 1-month postpartum
		Safety	Intensive care admission as observed by study staff	Recruitment to 1-month postpartum
		Safety	Need for blood transfusion, as observed by study staff	Recruitment to 1-month postpartum

		Safety	Delayed Adverse Events as detected by open questioning by study staff	Each scheduled visit (4 weeks post-intervention (for both IV iron and commencement of oral iron), 36 weeks, at delivery, 4 weeks postpartum
		Exploratory	Hospitalisation- any unplanned admission to hospital beyond usual postpartum discharge procedures, as observed by study staff	Following delivery
	Morbidity	Primary	Birth weight (as a continuous variable) using infant scales	Within 24 hours of birth
Neonate	Physical examination and anthropometry	Secondary	Low birth weight (birth weight <2500g) as a dichotomous variable	Within 24 hours of birth
		Exploratory	Gestational age (based on baseline ultrasound dating of pregnancy) adjusted birth weight	<24 hours following birth
		Secondary	Small for gestational age as a dichotomous variable (<10th centile)	<24 hours following birth
		Secondary	Gestation duration based on the calculated duration of gestation, using dating at baseline ultrasound examination to date of actual delivery	Delivery visit
		Secondary	Premature birth – neonate born prior to 37 completed weeks of gestation (including 36weeks and 6 days), based on gestation duration	Delivery visit
		Exploratory	Haemoglobin of venous cord blood by an automated analyser	Delivery
	Laboratory procedures	Secondary	Haemoglobin as measured on venous blood via automated analyser	1-month postpartum

		Exploratory	Incidence of cord blood parasitaemia at delivery based on blood film microscopy	Delivery
		Exploratory	Ferritin by serum ferritin	1 month of age
		Exploratory	Cord ferritin by serum ferritin	Delivery
		Secondary	Abortion - pregnancy loss before 28 completed weeks of gestation, as reported by the patient or based on clinical records, or as observed by study staff	<28 weeks' gestation
	Adverse events	Secondary	Stillbirth – defined as the birth of a baby showing no signs of life after 28 weeks of gestation (>28 weeks), as reported by the patient, based on clinical records, or as observed by study staff	>28 weeks' gestation
		Safety	Neonatal mortality, as observed by study staff/ clinical notes	Death of a child in the first month of life
		Safety	Neonatal intensive care admission as observed by study staff	Birth to 1-month postpartum
		Safety	Neonatal intensive care admission as observed by study staff	Birth to 1-month postpartum
