# Supplementary material for: Protocol for a multicentre, parallel-group, open-label randomised controlled trial comparing ferric carboxymaltose with the standard of care in anaemic Malawian pregnant women: the REVAMP trial
Source: BMJ Open. 2021 Nov 23;11(11):e053288. doi: 10.1136/bmjopen-2021-053288 (PMC8611444; doi:10.1136/bmjopen-2021-053288)
Supplement: Author's manuscript [file bmjopen-2021-053288.draft_revisions.pdf]

BMJ Open is committed to open peer review. As part of this commitment we make the peer review history of every article we publish publicly available.

When an article is published we post the peer reviewers' comments and the authors' responses online. We also post the versions of the paper that were used during peer review. These are the versions that the peer review comments apply to.

The versions of the paper that follow are the versions that were submitted during the peer review process. They are not the versions of record or the final published versions. They should not be cited or distributed as the published version of this manuscript.

BMJ Open is an open access journal and the full, final, typeset and author-corrected version of record of the manuscript is available on our site with no access controls, subscription charges or pay-per-view fees (http://bmjopen.bmj.com).

If you have any questions on BMJ Open's open peer review process please email info.bmjopen@bmj.com

BMJ Open

Protocol for a multicentre, parallel-group, open-label randomised controlled trial comparing ferric carboxymaltose with the standard of care in anaemic Malawian pregnant women – The REVAMP trial

Journal:	*BMJ Open*
Manuscript ID	bmjopen-2021-053288
Article Type:	Protocol
Date Submitted by the Author:	10-May-2021

Complete List of Authors:	Mwangi, Martin; Training and Research Unit of Excellence (TRUE), Nutrition and Infectious Diseases; University of Malawi College of Medicine, School of Public Health and Family Medicine, Department of Public Health<br>Mzembe, Glory; Training and Research Unit of Excellence (TRUE), Nutrition and Infectious Diseases; University of Malawi College of Medicine, School of Public Health and Family Medicine, Department of Public Health<br>Moya, Ernest; Training and Research Unit of Excellence (TRUE), Nutrition and Infectious Diseases; University of Malawi College of Medicine, School of Public Health and Family Medicine, Department of Public Health<br>Braat, Sabine ; University of Melbourne School of Population and Global Health, Centre for Epidemiology and Biostatistics; The University of Melbourne Melbourne Medical School, Department of Infectious Diseases<br>Harding, Rebecca; The Walter and Eliza Hall Institute of Medical Research, Population Health and Immunity Division<br>Robberstad, Bjarne; University of Bergen, Department of Global Public Health and Primary Care<br>Simpson, Julie; University of Melbourne School of Population and Global Health, Centre for Epidemiology and Biostatistics<br>Stones, William; University of Malawi, Obstetrics & Gynaecology<br>Rogerson, Stephen; University of Melbourne, Department of Medicine at the Peter Doherty Institute for Infection and Immunity; The University of Melbourne Melbourne Medical School, Department of Infectious Diseases<br>Biselele, Kabeya; Zomba Central Hospital<br>Chinkhumba, Jobiba; University of Malawi College of Medicine, Malaria Alert Centre<br>Larson, Leila; University of South Carolina Arnold School of Public Health, Department of Health Promotion, Education, and Behavior<br>Ataíde, Ricardo; The Walter and Eliza Hall Institute of Medical Research, Population Health and Immunity Division; The University of Melbourne, Department of Infectious Diseases<br>Phiri, Kamija; Training and Research Unit of Excellence (TRUE); University of Malawi College of Medicine, School of Public Health and Family Medicine, Department of Public Health<br>Pasricha, Sant-Rayn; Walter and Eliza Hall Institute of Medical Research, Population Health and Immunity Division; The Royal Melbourne Hospital, Diagnostic Haematology and Clinical Haematology, The Peter MacCallum

	Cancer Centre
Keywords:	Anaemia < HAEMATOLOGY, Nutrition < TROPICAL MEDICINE, Epidemiology < TROPICAL MEDICINE, Clinical trials < THERAPEUTICS, PUBLIC HEALTH, Epidemiology < INFECTIOUS DISEASES

SCHOLARONE™
Manuscripts

I, the Submitting Author has the right to grant and does grant on behalf of all authors of the Work (as defined in the below author licence), an exclusive licence and/or a non-exclusive licence for contributions from authors who are: i) UK Crown employees; ii) where BMJ has agreed a CC-BY licence shall apply, and/or iii) in accordance with the terms applicable for US Federal Government officers or employees acting as part of their official duties; on a worldwide, perpetual, irrevocable, royalty-free basis to BMJ Publishing Group Ltd ("BMJ") its licensees and where the relevant Journal is co-owned by BMJ to the co-owners of the Journal, to publish the Work in this journal and any other BMJ products and to exploit all rights, as set out in our licence.

The Submitting Author accepts and understands that any supply made under these terms is made by BMJ to the Submitting Author unless you are acting as an employee on behalf of your employer or a postgraduate student of an affiliated institution which is paying any applicable article publishing charge ("APC") for Open Access articles. Where the Submitting Author wishes to make the Work available on an Open Access basis (and intends to pay the relevant APC), the terms of reuse of such Open Access shall be governed by a Creative Commons licence – details of these licences and which Creative Commons licence will apply to this Work are set out in our licence referred to above.

Other than as permitted in any relevant BMJ Author's Self Archiving Policies, I confirm this Work has not been accepted for publication elsewhere, is not being considered for publication elsewhere and does not duplicate material already published. I confirm all authors consent to publication of this Work and authorise the granting of this licence.

For peer review only

Protocol for a multicentre, parallel-group, open-label randomised controlled trial comparing ferric carboxymaltose with the standard of care in anaemic Malawian pregnant women – The REVAMP trial

Authors

Martin N Mwangi[1,2], Glory Mzembe[1,2], Ernest Moya[1,2], Sabine Braat[7,8], Rebecca Harding[4], Bjarne Robberstad[5], Julie Simpson[8], William Stones[2], Stephen Rogerson,[6,7] Kabeya Biselele[12], Jobiba Chinkhumba[3], Leila Larson[9], Ricardo Ataíde[4, 7], Kamija S Phiri[1,2], Sant-Rayn Pasricha[4,10,11]

Institutions *(Full name, department, institution, city and country of all co-authors)*

1. Training and Research Unit of Excellence (TRUE), 1 Kufa Road, P.O. Box 30538, Chichiri, BT3, Blantyre, Malawi.
2. School of Public Health and Family Medicine, Department of Public Health, College of Medicine, University of Malawi, Private Bag 360, Chichiri, BT3, Blantyre, Malawi.
3. Malaria alert centre, College of Medicine, Blantyre, Malawi
4. Population Health and Immunity Division, The Walter and Eliza Hall Institute of Medical Research, 1G Royal Parade, Parkville, Melbourne 3052, Australia.
5. Department of Global Public Health and Primary Care, University of Bergen, Bergen, Norway
6. Department of Medicine at the Peter Doherty Institute for Infection and Immunity, University of Melbourne, Melbourne, Australia.
7. Department of Infectious Diseases, Melbourne Medical School, The University of Melbourne, Melbourne, Australia
8. Centre for Epidemiology and Biostatistics, Melbourne School of Population and Global Health, The University of Melbourne, Melbourne, Australia

For peer review only

9. Department of Health Promotion, Education, and Behavior, Arnold School of Public Health, University of South Carolina, Columbia, South Carolina, USA
10. Diagnostic Haematology, The Royal Melbourne Hospital; and Clinical Haematology, The Peter MacCallum Cancer Centre and The Royal Melbourne Hospital, Parkville VIC 3050 Australia
11. Department of Medical Biology, Faculty of Medicine, Dentistry and Health Sciences, The University of Melbourne
12. Zomba Central Hospital, Department of Obstetrics and Gynecology, P.O. Box 21, Zomba, Malawi.

Protocol registry number: ACTRN12618001268235

Corresponding author:

Martin N. Mwangi, P.O. Box 30538, Chichiri, BT3, Blantyre, Malawi. Email: mmwangi@true.mw

Word count: 4,521 words. (Abstract) 299 words. *(excluding title page, abstract, references, figures and tables)*

For peer review only

ABSTRACT

Introduction: Anaemia in pregnancy remains a critical global health problem, affecting 46% of pregnant women in Africa and 49% in Asia. Oral iron therapy requires extended adherence to achieve correction of anaemia and replenishment of iron stores. Ferric carboxymaltose (FCM) is a recently established intravenous iron formulation associated with substantial advantages in safety, speed of delivery and total dose deliverable in a single infusion. We aim to determine whether FCM given once during the second trimester of pregnancy compared to standard oral iron distributed through routine antenatal services is effective and safe for treatment of moderate to severe maternal anaemia in sub-Saharan Africa.

Methods and analysis: REVAMP is a two-arm confirmatory individually-randomised controlled trial set in Blantyre and Zomba districts in Malawi. The trial will randomise 862 women in the second trimester of pregnancy with a capillary haemoglobin concentration below 10.0g/dL. The study comprises two arms: (a) intravenous ferric carboxymaltose (20mg/kg up to 1000mg) given once at randomisation, and (b) standard of care oral iron (65mg elemental iron twice daily) for 90 days (or the duration of pregnancy, whichever is shorter) provided according to local health care practices. Both arms receive sulfadoxine-pyrimethamine (SP) as Intermittent Preventive Treatment in pregnancy (IPTp). The primary outcome is the prevalence of anaemia (Hb<11.0g/dL) at 36 weeks' gestation. Secondary outcomes include birth weight, gestation duration, and safety outcomes, including clinical malaria, serious perinatal events and postpartum haematologic and health-related outcomes in the mother and child.

Ethics and dissemination: Ethical approval was granted by the Research Ethics Committee (COMREC P.02/18/2357) in Malawi and the Human Research Ethics Committee (WEHI: 18/02), Melbourne, Australia. The protocol is registered with the Australian and New Zealand Clinical Trials Registry

For peer review only

(ACTRN12618001268235). The results will be shared with the local community that enabled the research, and also to the international fora.

Keywords: Anaemia, Iron Deficiency, Intravenous Iron, ferric carboxymaltose (FCM), Pregnancy, Malawi, randomised controlled trial

Data Statement

All relevant data are within the paper and its supporting information files.

Protocol version

REVAMP Trial protocol version 5.0 dated 21 November 2020

Ethics approval

College of Medicine Research Ethics Committee (COMREC P.02/18/2357) in Malawi and the Walter and Eliza Hall Institute of Medical Research Human Research Ethics Committee (WEHI: 18/02), Melbourne, Australia.

Trial Sponsor

Director - Research Support Centre

College of Medicine, Private Bag 360, Chichiri, Blantyre 3, Malawi

Email: rscdirector@medcol.mw

Roles and responsibilities of sponsor and funder:

The sponsor and funder had no role in study design. They will have no role nor ultimate authority in the collection, management, analysis, and interpretation of data; writing of the report; and the decision to submit the report for publication.

For peer review only

ARTICLE SUMMARY

Strengths and limitations of this study

1. Trial setting: The trial is conducted in a low-income sub-Saharan African setting where there is a high prevalence of anaemia in pregnancy, low birth weight and *P. falciparum* malaria. The trial will provide critical new data on the effectiveness and safety of intravenous iron in a context where a) anaemia and iron deficiency rates are high, b) malaria is prevalent and causes anaemia, and c) iron interventions may increase malaria infection rates. In these settings, needs for anaemia control are critical.
2. Participant screening: Eligibility for inclusion is determined by moderate or severe anaemia detected via capillary haemoglobin estimation, which at present is a convenient and available method for assessing anaemia status that could be deployed in the high-throughput health centre setting; our results will thus provide direct information on the impact of this highly feasible screening and treatment approach for antenatal anaemia.
3. Use of ferric carboxymaltose: this is the first trial in a low-income field setting to use a recently established intravenous iron formulation for the treatment of anaemia in pregnancy; previous trials in low-income settings have used older formulations such as iron sucrose and iron dextrans, which may have a higher risk of adverse events and take longer to infuse, and thus are suboptimal for routine deployment in the health centre setting.
4. Outcomes: our trial includes a critical primary outcome of anaemia before delivery, as well as key secondary outcomes to assess the benefit of the trial intervention for mother and neonate, and detailed safety assessments using active and passive data collection.
5. Sample size: this is the largest trial of ferric carboxymaltose in pregnancy. It is also among the largest trials of ferric carboxymaltose (and intravenous iron) ever conducted. The study will thus provide critical effectiveness and safety information on this drug's role in pregnancy globally.

For peer review only

INTRODUCTION

Anaemia during pregnancy remains a critical global health problem. Almost 40% of pregnant women worldwide are anaemic, including 46% of pregnant women in Africa and 49% in Asia.[1] Anaemia in pregnancy is very common and mostly results from iron deficiency and is associated with critical risks for both mother (e.g. life-threatening complications of postpartum haemorrhage) and child (especially prematurity and low birth weight,[2] which are associated with increased risk of neonatal and infant mortality,[3] and reduced iron stores in infancy with increased risk of subsequent anaemia and impaired development).[4] Severe anaemia in pregnancy is associated with a significantly increased risk of maternal mortality.[5] Worldwide, 15%-20% of births (>20 million annually, including 13% in sub-Saharan Africa) are low birthweight. At the same time, each year, over 1 million children die from preterm birth complications, making prevention and treatment of antenatal anaemia a crucial component of efforts to reduce low birth weight.

Systematic reviews confirm likely benefits from iron on maternal outcomes, including anaemia (70% reduction) and trends towards favourable infant outcomes, including increased birth weight and extended gestation duration.[6] A trial of oral iron compared with placebo in Kenyan pregnant women confirmed a benefit from iron on birth weight and gestation duration.[7] Global recommendations for managing anaemia in pregnancy in lower and middle income countries (LMICs) currently advise that women be treated with high-dose daily oral iron (120 mg of elemental iron) supplementation for three months[8 9]. However, oral iron is often poorly tolerated due to gastrointestinal adverse effects,[10] limiting adherence. Delivery of iron during pregnancy requires high-quality performance of the primary health system. In Malawi, 10.6% of women were reported to take no iron during pregnancy, and 37.1% take less than 60 doses, with only 33.4% taking the recommended 90 or more doses.[11] Even when delivered, oral iron frequently fails to correct anaemia. For example, 61% of Gambian pregnant women who received iron at week 20 of gestation – when their Hb was lower than 10.0g/dL - still had a Hb<10.0g/dL at 30 weeks' gestation.[12] Finally, women may only present for their first antenatal visit

For peer review only - http://bmjopen.bmj.com/site/about/guidelines.xhtml

late in the second trimester, curtailing the time available to optimise iron stores with oral iron to improve foetal development and risks associated with delivery; for example, in Malawi, fewer than 30% of pregnant women attend before the sixteenth week of gestation.[13]

Over the past decade, there have been dramatic improvements in parenteral (intravenous) iron therapeutics. Ferric carboxymaltose (FCM) is a widely used formulation that can be administered in a short period (15 min) and at large doses (up to 1000 mg) in a single infusion[14 15]. FCM has revolutionised the treatment of iron-deficiency anaemia in high-income country settings and is widely used in outpatient settings, emergency departments, and primary care.[16] In pregnancy, intravenous iron has been reported to be superior to oral iron in improving haemoglobin concentrations and may even increase birth weight.[17] Intravenous iron is increasingly recommended as a suitable first-line option for women with moderate or severe iron-deficiency anaemia beyond the first trimester of pregnancy.[18]

It remains challenging to directly measure iron status in the field, as biomarkers generally require analysis using centralised laboratory infrastructure. In contrast, point-of-care measurement of capillary haemoglobin is a feasible field-friendly testing strategy that can be deployed with existing technology, of which HemoCue point-of-care devices are a good example. Screen-and-treat approaches for anaemia in pregnancy must rely on haemoglobin measurement until point-of-care assays for iron status become more widely available.

FCM presents a practical opportunity for rapid correction of moderate and severe antenatal anaemia in a single dose, potentially improving maternal and neonatal clinical outcomes for pregnant women. It is, therefore, urgent to test this therapy in a low-income field setting where the burden of anaemia-related disease is heaviest. In Malawian pregnant women with moderate or severe anaemia during the second trimester of pregnancy, we aim to evaluate whether a single dose of intravenous ferric

For peer review only - http://bmjopen.bmj.com/site/about/guidelines.xhtml

For peer review only

carboxymaltose is superior to standard-of-care oral iron.

METHODS AND ANALYSIS

Patient and Public Involvement

We held discussions with national policy stakeholders during the study planning stage, including the Ministry of Health. We discussed how the research might align with national research and health service priorities. Local community engagement was done via public meetings; the potential participants were first involved in the study's design during these meetings. We discussed the study with the traditional leaders, including traditional authorities (TAs), group village headmen (GVH), the village development committee (VDC), and ward councillors (political figures). The GVH were requested to cascade the information to the village chiefs who took the information to the community. Health workers were accessed via the District Health Offices in Blantyre and Zomba districts. Health workers from the participating health facilities, including the health surveillance assistants (HSAs) who directly work with the community, were informed about the proposed research and discussions on priorities beneficial to the community were discussed. The public, potential participants, and health workers identified malaria and anaemia as major public health issues in the community. Women outlined their experiences with iron supplements during their past pregnancies and identified the development of tolerable iron formulations as a research priority. Dissemination of findings at the national and community level will follow the schema used during study design and inception as outlined above.

For peer review only

Trial objectives

The primary objective of the trial is to determine whether, in Malawian women in the second trimester of pregnancy with moderate or severe anaemia, a single dose of FCM up to 1000mg is superior to standard-of-care (i.e. oral iron provided through local health services), in reducing maternal anaemia before delivery (at 36 weeks' gestation).

For peer review only - http://bmjopen.bmj.com/site/about/guidelines.xhtml

The secondary objectives are:

During pregnancy and up to one month postpartum, to determine the effects of FCM (compared with standard-of-care) on:

- Maternal haemoglobin concentration and iron status (measured through iron biomarkers),
- Critical neonatal outcomes including birth weight (low birth weight), gestation duration (prematurity), small for gestational age and other perinatal outcomes, and
- Maternal and neonatal adverse events, including infection episodes, serious maternal complications, and hypophosphatemia.

Exploratory objectives up to one month postpartum for subsequent hypothesis generation are related to maternal cognition, depression, and fatigue, as well as costs of health care.

Study design

REVAMP is a multicentre, open-label, two-arm, parallel-group randomised controlled trial (RCT). An open-label (rather than blinded) design was selected as it was considered unfeasible to deliver the placebo intravenous infusions to Malawian pregnant women in this field-trial context (the drug is dark-coloured). The trial is recruiting pregnant women in their second trimester and following them until one month postpartum, after which we will report on the primary and secondary objectives. Extended follow-up of mothers and infants to 12-months postpartum is planned, as is a range of exploratory economic, biological and clinical outcomes. These analyses are beyond the scope of this protocol, as are the exploratory outcomes collected up to one month postpartum, and they will be reported separately. The trial design is summarised in **Figure 1**.

Study settings and participants

For peer review only

In Malawi, the average gestation age at the first antenatal clinic (ANC) visit is 22 weeks, with 61% presenting before 24 weeks. The 2015-16 DHS survey estimated a prevalence of anaemia (Hb < 11.0g/dL) in pregnant women in Malawi at 45.1%, including 22.4% of women with moderate or severe anaemia.[11] The prevalence of low birth weight in Malawi was around 14% in 2015.[19] Malawi is endemic for malaria and the prevalence of *Plasmodium spp.* parasitaemia at the first ANC visit in the study site exceeds 15%.[20] About 87% of women consume at least one dose of intermittent preventive treatment during pregnancy (IPTp), but this falls to 30% for women taking three or more doses.[11]

The REVAMP study has two major research sites namely Limbe Health Centre and Zomba Central Hospital located in Blantyre and Zomba districts respectively, in Southern Malawi. The two major sites act as the study coordination centres in the respective districts. The Blantyre site serves as the base for five urban health centres/antenatal clinics, whilst the Zomba site serves as the base for nine (four urban and five rural) health centres/antenatal clinics. Women are screened at the antenatal clinics and referred to the coordinating research site for enrolment, treatment and follow-up visits. Deliveries occur in government-operated birth suites attached to the study site with referral to a district hospital where required for obstetric indications.

For peer review only

Eligibility criteria

Women are only randomised if they fulfil all inclusion criteria and none of the exclusion criteria.

Inclusion criteria:

1. Confirmed singleton pregnancy at 13-26 weeks' gestation
2. Moderate to severe anaemia (capillary haemoglobin <10.0g/dL measured by HemoCue 301+) not clinically deemed to require an immediate blood transfusion

3. Negative malaria parasitaemia at screening and no positive malaria rapid diagnostic test within the previous seven days
4. Resident in the study catchment area of Blantyre and Zomba district
5. Plan to deliver at a health facility
6. Written informed consent, including assent if <18 years old (consent of a parent or guardian plus the participant's assent).

Exclusion criteria:

1. Hypersensitivity to any of the study drugs
2. Clinical symptoms of malaria or positive malaria rapid diagnostic test within the previous seven days, or symptoms of bacterial infection, at screening
3. Any condition requiring hospitalisation or serious concomitant illness
4. Chronic illness that may adversely affect foetal growth and viability
5. Severe anaemia clinically judged as requiring a blood transfusion (usually Hb <5.0g/dL)
6. Pre-eclampsia at screening (new onset of hypertension, proteinuria and swelling in the legs, feet, and hands).

Trial Interventions

Participants are randomly assigned to receive one of the following interventions:

1. **Intravenous iron**: ferric carboxymaltose 20mg/kg up to 1000mg, diluted in normal saline and given over 15 minutes, once after randomisation. The study clinician administers the drug, and women remain under observation for 45 minutes after drug administration. Women receiving FCM are treated in rooms equipped with a trolley containing adrenaline, hydrocortisone, intravenous fluids and antihistamines, airway equipment and an oxygen concentrator. Staff are trained in the management of allergic reactions.

For peer review only

2. **Standard-of-care - Oral iron treatment course**: oral iron- 200mg ferrous sulphate (approx. 65mg elemental iron) twice daily for 90 days or the remaining duration of pregnancy, whichever is shorter. Oral iron is provided by health workers using the same education messaging and similar packaging as provided by the routine health system to reflect real-life health service conditions.

Following national guidelines, both groups receive IPTp with 1500 mg sulfadoxine and 75 mg pyrimethamine (SP), as three tablets of SP of fixed dose (500mg/25mg) at least three times during pregnancy, at least four weeks apart. The IPTp-SP course at four weeks' post-randomisation is directly observed by study staff. HIV positive women in Malawi are not offered SP because they are already on cotrimoxazole prophylaxis. The baseline dose is also directly administered by study staff if the participant did not receive it through the health service. All participants also receive an insecticide-treated bed net.

Randomisation, allocation concealment, and blinding

Participants are randomly allocated to one of the two treatments arms with 1:1 allocation via a computer-generated randomisation schedule of randomly permuted blocks stratified by research site (Blantyre, Zomba) to achieve a balance between the arms within each site. The randomisation list was generated by an independent statistician at the University of Melbourne (Australia).

Individual participant codes were pre-packed in sealed envelopes by an independent researcher not associated with the study and held securely at the research sites. Eligible participants who meet all inclusion/exclusion criteria are sequentially allocated participant identification numbers within the research site, and their allocation to study group is revealed after opening the corresponding sealed envelope. Although the trial is open-label, laboratory scientists measuring haemoglobin concentration, midwives collecting birth outcome data, and investigators and researchers in Australia

For peer review only - http://bmjopen.bmj.com/site/about/guidelines.xhtml

(including data managers and statisticians in Melbourne) are blinded to the treatment allocation during the conduct of the trial until the database is locked and ready for unblinding.

Recruitment and Visits

Table 1 shows the schedule of activities per visit for the mother, and **Table 2** shows the schedule of activities per visit for the neonate.

For peer review only

For peer review only - http://bmjopen.bmj.com/site/about/guidelines.xhtml

Table 1: Planned activities per visit for mothers

**Protocol activity**	**Visit 0**	**Visit 1**	**Visit 2**	**Visit 3**[j]	**Visit 4**	**Visit 5 – 6**[j]	**Visit 7**	**Visit 8**
	Day -7 to 0	Day 0	Day 28 ±2 days	34 wks. gestation ±2 days	36 wks. gestation ±2 days	38-40 wks. gestation ± 2 days	Delivery + 1 day	Day 28 postpartum ±2 days
Location of visit	*Antenatal Clinic*	*Research Site*[a]	*Research Site*[a]	*Home*[b]	*Research Site*[a]	*Home*[b]	*Research Site*[a]	*Research Site*[a]
Informed consent process for pre-screening	X							
Pre-screening	X							
Detailed Informed consent process	X	X						
Screening		X						
Medical & obstetric history		X						
Demographics		X						
Maternal physical examination[d]		X[h]	X[i]		X[i]		X[h]	X[i]

For peer review only

Ultrasound scan **(Fetal Biometry)**[d]		X	X		X			
Randomise participant		X						
Administer treatment		X						
Intermittent preventive treatment[c]		X	X		X			
Laboratory procedures								
Capillary haemoglobin[d]	X	X	X	X	X	X	X	X
Full blood count[d]		X	X		X		X	X
Malaria diagnostics[d, e]	X	X	X		X		X	X
Serum for iron markers tests[f]		X	X		X		X	X
Serum for inflammatory markers tests[g]		X	X		X		X	X
Phosphate		X	X		X		X	X
Adverse events[d]		X	X		X		X	X
Morbidities								X
Missed visits[d]			X	X	X	X	X	X
End of study			X	X	X	X	X	X

For peer review only

[a] Visit conducted at the research site/health facility; [b] Visit conducted at the participant's home; [c] Assessing if intermittent preventive treatments sulphadoxine pyrimethamine and albendazole were given; [d] Protocol activities are also collected at any unscheduled visits; [e] Malaria diagnostics include malaria rapid diagnostic test, malaria microscopy, [f] Serum for iron markers tests include serum ferritin; [g] Serum for inflammatory markers tests include C-reactive protein and alpha-1 glycoprotein; [h] Complete maternal physical examination includes general appearance, throat, neck, thyroid, musculoskeletal, skin, lymph nodes, extremities, pulses, pulmonary, cardiac, abdominal, and neurological examination; [i] Limited maternal physical examination includes general appearance, brief pulmonary, cardiac, abdominal and neurological examination. [j] Since the opening of the trial, visits 3, 5 and 6 have been suspended since April 2020 due to the risk of exposing communities to COVID-19 during home visits.

For peer review only

Table 2: Details of planned activities per visit for neonates

**Protocol activity**	**Visit 7**	**Visit 8**
	Delivery +1 day	Day 28 Postpartum ±2 days
Location of visit	*Research Site[a]*	*Research Site[a]*
Pregnancy outcome	X	
Physical examination and anthropometry [b, c]	X[f]	X[g]
Laboratory procedures		
Capillary haemoglobin	X[h]	
Full blood count[b]	X[h]	X
Malaria diagnostics[d]	X[h]	X
Serum for iron markers tests[e]	X[h]	X
Vaccination and Vit A supplementation status		X
Adverse events[b]	X	X
Morbidities		X
Missed visits[b]		X
End of study	X	X

[a] Visit done at the research site/ health facility; [b] Protocol activities are also collected at any unscheduled visits; [c] Anthropometry includes assess the infant's weight, length, and head circumference; [d] Malaria diagnostics include malaria rapid diagnostic test, malaria microscopy, malaria filter paper for polymerase chain reaction and histology at delivery; [e] Serum for iron markers tests include serum ferritin; [f] The complete physical examination includes general appearance, throat, neck, thyroid, musculoskeletal, skin, lymph nodes, extremities, pulses, pulmonary, cardiac, abdominal, and neurological examination [g] The limited examination includes general appearance, brief pulmonary, cardiac, abdominal and neurological examination; [h] These assessments are collected via cord blood.

For peer review only - http://bmjopen.bmj.com/site/about/guidelines.xhtml

Screening and enrolment of participants

Study activities are detailed in Tables 1 and 2.

Pre-screening and screening, visit 0 (day -7 to 0)

Women attending their first antenatal visit at ANCs are given general information (aims and procedures) about the study by a trained research nurse and invited to consent to pre-screening procedures. As the ANC nurse cares for attendees, the research nurse pre-screens potential participants by noting apparent gestation age based on clinical examination and last menstrual period and then tests for anaemia with capillary Hb (HemoCue 301+, Angelholm Sweden) and *Plasmodium* parasitaemia by rapid diagnostic test (SD Bioline Malaria AG P.F/PAN, Standard Diagnostics, Inc.). Potentially eligible participants receive further information about the study. Willing participants are referred to the research site for additional evaluation, full consenting procedure and recruitment.

Enrolment, visit 1 (day 0)

As presented in Table 1, at the health facility, potentially eligible participants undergo ultrasound scanning (within two days from the initial pre-screening visit) to confirm singleton pregnancy and determine gestation age (i.e. crown-rump length for less than or equal to 16 weeks, femur length and head and abdominal circumference for more than 16 weeks). Written informed consent is then obtained for participation in the trial. For consenting participants, a physical examination is conducted, and baseline data are collected, comprising demographic information and medical and obstetric history. Following the collection of baseline clinical data, samples of both capillary and venous blood are collected and then participants are randomised. For women randomised to the ferric carboxymaltose arm of the trial, an intravenous cannula is inserted under aseptic precautions, and a trained nurse administers the study drug according to standard procedures and under the supervision of the study clinician. Women randomised to the standard-of-care arm of the trial are provided with

For peer review only - http://bmjopen.bmj.com/site/about/guidelines.xhtml

a full course of iron tablets, together with information delivered according to a standardised script reflecting ANC practice.

Subsequent study visits

Visit 2 (28 (±2) days after enrolment into the study): Participants receive a physical examination and an ultrasound scan before being asked for a venous blood sample (Table 1). A capillary sample is collected for assessment of *Plasmodium* parasitaemia. The participants are asked to continue their regular ongoing ANC visits through their local health centre.

Visit 3; visits 5-6 (34 weeks' gestation ±2 days; 38-40 weeks' gestation ±2 days): Participants are visited in their home at 34 weeks' gestation, and every two weeks from 38 weeks' gestation until delivery. A research nurse measures capillary Hb. Where the participant belongs to the standard-of-care trial-arm, the research nurse attempts to assess adherence. Notably, since the opening of the trial, fieldwork has been affected by the COVID-19 pandemic. Because of the risks of study workers introducing COVID-19 into remote villages, home visits were removed from the trial protocol after April 2020.

Visit 4 (36 weeks' gestation/pre-delivery ±2 days): Procedures are similar to those for Visit 2 (28-days post-treatment). This visit includes the assessment of venous haemoglobin for the primary outcome.

Visit 7 (Delivery +1 day): The study provides 24-hour cover of the study research sites' delivery suites. All participants are asked to return to the research site for delivery (unless a high-risk pregnancy requires delivery at the tertiary referral hospital). Standardised delayed cord clamping procedures are instituted. Participants delivering at home or at other health facilities are encouraged to attend the research site for assessment within 24 hours. Venous blood samples are collected during labour. Apgar scores are recorded immediately after delivery, and the newborn undergoes a full physical examination, including measurement of birth weight, length, head circumference, and assessment for congenital malformations. The type of birth and occurrence of perinatal complications (including

For peer review only

For peer review only - http://bmjopen.bmj.com/site/about/guidelines.xhtml

haemorrhage or need for blood transfusion) are recorded. Placental blood is drawn, and a sample of placental tissue is collected and stored in buffered formalin for subsequent histological evaluation.

Visit 8 (28 days postpartum ±2 days): Participants return to the research site together with their infants for a detailed medical examination of both mother and infant and collection of blood samples.

Unscheduled visit: Participants are asked to attend the research site when symptomatically unwell. They are managed according to national standard treatment guidelines by a trained health care provider. Blood samples for malaria RDT (and microscopy if RDT positive) and blood culture are taken if clinically indicated. The clinical diagnosis of the unscheduled visit is recorded in the participant's record. Where a participant attends another health facility or antenatal clinic, the research team extracts the missed unscheduled visit notes from the participants health book commonly known as a health "passport" in Malawi. Usually, clinical investigations and medications are indicated in the health book and can be extracted by the research team during the next scheduled visit.

Data Monitoring Committee (DMC)

An independent DMC has been set up to review on a regular basis, safety and efficacy data of the ongoing trial. The DMC is comprised of international experts in clinical trials, obstetrics, epidemiology and statistics. In the advent of clear evidence of effectiveness or harm, the DMC will recommend to the sponsor and investigators whether to continue, modify, or terminate the trial on ethical grounds.

For peer review only

Outcomes

The primary outcome is the prevalence of maternal anaemia before delivery, defined as a venous haemoglobin concentration less than 11.0g/dL at 36 weeks' gestation.

Secondary outcomes assessing effectiveness in the mother are anaemia at four weeks' post-randomisation and one month postpartum, haemoglobin concentration, iron deficiency (ferritin<15mg/L adjusted for C-reactive protein), iron deficiency anaemia, and serum ferritin concentration all at four weeks post-randomisation, 36 weeks' gestation, and one month postpartum.

Effectiveness in the neonate is assessed using the outcome of mean birthweight (measured in grams within 24 hours of birth) and the secondary outcomes low birth weight (birth weight less than 2500g), gestation duration (weeks), gestational-age-specific birth weight, small for gestation age, fetal loss (pregnancy loss before 28 completed weeks' gestation), stillbirth (birth of a child showing no signs of life after 28 weeks' gestation), and preterm births (neonate born before 37 completed weeks of gestation), haemoglobin concentration at 1-month postpartum and infant growth (weight for age, length for age, weight for length, head circumference) at one month postpartum.

Outcomes assessing safety in the mother are (serious) adverse events related to administration of FCM (events occurring during the enrolment visit), (serious) adverse events (within 14 days of randomisation, antenatal and postpartum reported separately), all-cause sick visits to the clinic (antenatal and postpartum reported separately), diarrhoea-related visits to the clinic (antenatal and postpartum reported separately), clinical malaria-specific visits to the clinic (antenatal and postpartum reported separately), placental *P. falciparum* (delivery), *P. falciparum* parasitaemia (at four weeks' post-randomisation and 36 weeks' gestation), hypophosphatemia (biochemical) (at four weeks' post-randomisation and 36 weeks' gestation), inflammation (elevated C-reactive protein) (at four weeks' post-randomisation and 36 weeks' gestation), and severe medical events including haemorrhage, sepsis, shock, need for transfusion, ICU admission, or mortality.

For peer review only - http://bmjopen.bmj.com/site/about/guidelines.xhtml

Safety in the infant is assessed using (serious) adverse events, all-cause visits to the clinic, infection-related visits to the clinic, diarrhoea-related visits to the clinic, clinical malaria-specific visits to the clinic, and *P. falciparum* parasitaemia by microscopy at one month postpartum.

In addition to the primary and secondary clinical outcomes listed above, which will support the reporting of the confirmatory RCT, data for many exploratory outcomes are being collected between enrolment and 12-months postpartum. These include assessing maternal cognition, mother-infant bonding, maternal depression and fatigue, infant neurodevelopment, infant diet and health economic data, including direct and indirect costs of health care. Also, women have given their consent for the collection of samples for future translational studies, including evaluation of the vaginal and faecal microbiome, maternal immune profiles, and effects of interventions directed towards *P. falciparum* biology.

Detection and reporting of Adverse Events and Serious Adverse Events

Non-serious Adverse Events (AEs) and serious adverse events (SAEs) are collected from the time consent is given until the participant completes the study (the final visit or withdrawal). These are detected either through spontaneous reports by the participant, unplanned visits to the research site or any of the participating health centres, observation by the study staff, and through standard questioning at each visit. All SAEs are reported to the sponsor and the appropriate ethics committee, whether or not considered causally related to the study drugs.

Sample size

The sample size calculation is based on the primary maternal outcome of the proportion of women with pre-delivery anaemia (defined as venous Hb<11.0g/dL at 36 weeks' gestation). A systematic review and meta-analysis identified a 50% reduction in anaemia prevalence in women receiving oral

For peer review only - http://bmjopen.bmj.com/site/about/guidelines.xhtml

iron.[21] In a cohort of pregnant women in the Gambia, we observed that 60% of pregnant women with Hb<10.0g/dL at 20 weeks' gestation who then receive iron interventions continue to have Hb<10.0g/dL by 30 weeks.[22] The pivotal Fer-ASAP trial (which compared FCM to oral iron in women with iron deficiency anaemia in high-income settings) demonstrated a 14% reduction in absolute anaemia prevalence compared with oral iron.[23] We hypothesise that routine iron supplementation will reduce anaemia prevalence from 100% to 60% (as seen in the Gambia,[22] and similar to data from Haider and colleagues [21]). We hypothesise that ferric carboxymaltose will result in a 10% absolute improvement in anaemia cure (to a prevalence of 50%). A total sample size of 862 pregnant women or 431 per group will provide 80% power to detect this difference, allowing for a 10% loss to follow-up and assuming a two-sided alpha of 5%. The sample size also has at least 80% power to detect an absolute difference between standard-of-care oral iron and ferric carboxymaltose of 100g in the neonatal outcome of birth weight (assuming a standard deviation of 450g and a two-sided alpha of 5%), similar to the effect size seen in a trial of Kenyan women receiving oral iron when compared to placebo.[7]

STATISTICAL ANALYSIS PLAN

A detailed statistical analysis plan will be finalised before unblinding of the database. Analyses will be performed where participants are classified according to their randomised intervention arm (i.e. intention to treat principle). An available case analysis will be performed for repeated time point outcomes (e.g. anaemia) and a complete-case analysis for single time point outcomes (e.g. birth weight). Anaemia will be analysed using a log-binomial regression model, including study participants as a random intercept to account for the multiple time points.

The model will include the standard-of-care (oral iron) group as the reference group. The primary maternal hypothesis will be evaluated by obtaining the estimate of the prevalence ratio of IV iron versus standard-of-care (oral iron), 95% confidence interval extracted at 36 weeks' gestation, and p-

For peer review only - http://bmjopen.bmj.com/site/about/guidelines.xhtml

value. Birthweight will be analysed by fitting a linear regression model. The primary neonatal hypothesis will be evaluated by estimating the absolute difference in mean birth weight between IV iron and standard-of-care (oral iron) along with a corresponding 95% confidence interval and p-value. Secondary repeated time point binary outcomes will be analysed similarly to anaemia, and secondary single time point continuous outcomes will be analysed similarly to birthweight. Secondary, single time point binary outcomes (e.g., sub-optimal pregnancy outcomes) will be analysed using a log-binomial regression model and secondary, multiple time point continuous outcomes (e.g., maternal haemoglobin concentration) will be analysed using a likelihood-based longitudinal data analysis model.[24] Appropriate transformations may be applied to the variables before fitting the model if considered skewed (e.g. ferritin). Additional analyses using multiple imputation will be performed to handle missing data. Results will be compared with the main analysis to investigate the findings' robustness to the missing data assumptions. Safety, including adverse events, infections and clinic visits, will be presented for the mothers and neonates, respectively. The proportion of study participants with at least one safety outcome will be compared between groups using a log-binomial regression model. A Poisson model with robust standard errors will be fitted if there is a non-convergence of the log-binomial model for efficacy or safety outcomes. Exploratory subgroup analyses (e.g., by parity, site, iron deficiency) will be performed for maternal and neonatal outcomes, irrespective of their findings. The analyses models for all study outcomes will adjust for the randomisation stratification variable of the site as a main effect.

For peer review only

Data Management

Data collected from the subjects are recorded in digital Case Report Forms (CRFs) using tablets. Relevant hard copy patient hospital files are scanned for reference and stored digitally, securely. An independent Data and Safety Monitoring Board has been established to regularly review the trial's

progress and blinded and unblinded results. The Research Support Centre at the College of Medicine performs independent monitoring of the study on behalf of the sponsor. No interim analysis is planned.

ETHICS AND DISSEMINATION

The College of Medicine Research Ethics Committee (COMREC), Blantyre, Malawi, and the Walter and Eliza Hall Institute of Medical Research Human Research Ethics Committee, Melbourne, Australia, approved this study. In addition, an evaluation was done by the Regional Ethics Committee West of Norway. They advised that the study was not subject to the Norwegian Health Research Act and that ethical review from this committee was not required. Important protocol modifications such as changes to eligibility criteria or outcomes are reported to the ethics committees, regulatory authorities in Malawi, investigators, trial participants, trial registries. Witnessed informed written consent in English or Chichewa language is obtained from each participant before conducting any study-related procedure. The study is conducted under The International Conference on Harmonisation of Technical Requirements for Registration of Pharmaceuticals for Human Use (ICH) guidelines for "good clinical practice" (GCP) and the Declaration of Helsinki. The results will be presented to and shared with the local community that hosted and enabled the research, and also to the international fora. We will publish in peer-reviewed scientific journals and report to relevant policymaking bodies such as the Malawi Ministry of Health.

DISCUSSION

Maternal anaemia prevalence is highest in sub-Saharan Africa and South Asia, and current approaches to control it are failing. This trial will provide high quality, African-based evidence for clinicians,

For peer review only

policymakers and donors on a role for modern IV iron for antenatal anaemia in low-income settings. This is the largest RCT program assessing new IV iron formulations in pregnancy and is of major international significance in developing new global guidelines for anaemia in pregnancy. The results of this study will be disseminated to local and national medical authorities, policymakers, and be disseminated to the global research community, technical agencies, and international government bodies via peer-reviewed journals and at international scientific fora.

Author Contributions:

MNM wrote the first draft of the paper. All authors read and approved the final manuscript.

Funding statement: This study is funded by the Bill & Melinda Gates Foundation OPP1169939.

Acknowledgements:

We acknowledge all the study participants for their willingness to participate in this study. We appreciate the dedication of the research staff in Blantyre, Zomba and Melbourne. We are grateful for the support we continue to receive from the District Health Offices in Blantyre and Zomba districts of Malawi. We acknowledge the collaboration with the Zomba Central Hospital, especially the office of the Hospital Director. We thank Dr Alinune Kabaghe and Dr Josephine Banda, who independently packed the trial drugs and delivered them to research sites. We also acknowledge the Dr Kamiza Histopathology Laboratory for analysing the placental samples.

Competing interests statement.

None to declare.

Word Count: 4,521

For peer review only

References

1. World Health Organization. The global prevalence of anaemia in 2011. . In: World Health Organization, ed. Geneva, 2015.
2. Haider BA, Olofin I, Wang M, et al. Anaemia, prenatal iron use, and risk of adverse pregnancy outcomes: Systematic review and meta-analysis. *BMJ (Online)* 2013;347(7916) doi: 10.1136/bmj.f3443
3. Premru-Srsen T, Verdenik I, Ponikvar BM, et al. Infant mortality and causes of death by birth weight for gestational age in non-malformed singleton infants: a 2002-2012 population-based study. *J Perinat Med* 2018;46(5):547-53. doi: 10.1515/jpm-2017-0103 [published Online First: 2017/06/11]
4. World Health Organization, United Nations Children's Fund, United Nations University. Iron Deficiency Anaemia: Assessment, Prevention, and Control. A guide for programme managers. In: Organization. WH, ed. Geneva, 2001.
5. Daru J, Zamora J, Fernandez-Felix BM, et al. Risk of maternal mortality in women with severe anaemia during pregnancy and post partum: a multilevel analysis. *Lancet Glob Health* 2018;6(5):e548-e54. doi: 10.1016/S2214-109X(18)30078-0 [published Online First: 2018/03/25]
6. Peña-Rosas JP, De-Regil LM, Garcia-Casal MN, et al. Daily oral iron supplementation during pregnancy. *Cochrane Database of Systematic Reviews* 2015;2015(7):1-527. doi: 10.1002/14651858.CD004736.pub5
7. Mwangi MN, Roth JM, Smit MR, et al. Effect of Daily Antenatal Iron Supplementation on Plasmodium Infection in Kenyan Women: A Randomized Clinical Trial. *JAMA* 2015;314(10):1009-20. doi: 10.1001/jama.2015.9496 [published Online First: 2015/09/09]
8. World Health Organization. Iron and folate supplementation. Integrated management of pregnancy and childbirth (IMPAC). . In: WHO. Geneva, 2006.
9. World Health Organization. Essential Nutrition Actions: Improving maternal, newborn, infant and young child health and nutrition. . In: WHO. Geneva, 2013.
10. Low MSY, Speedy J, Styles CE, et al. Daily iron supplementation for improving anaemia, iron status and health in menstruating women. *Cochrane Database of Systematic Reviews* 2016;2016(4) doi: 10.1002/14651858.CD009747.pub2
11. National Statistical Office (NSO) [Malawi], ICF International. Malawi Demographic and Health Survey 2015-16: Key Indicators Report. Zomba, Malawi, and Rockville, Maryland, USA.: NSO and ICF International., 2016.
12. Bah A, Pasricha SR, Jallow MW, et al. Serum hepcidin concentrations decline during pregnancy and may identify iron deficiency: Analysis of a longitudinal pregnancy cohort in the Gambia. *Journal of Nutrition* 2017;147(6):1131-37. doi: 10.3945/jn.116.245373
13. Mkandawire P. Gestational Age at First Antenatal Care Visit in Malawi. *Matern Child Health J* 2015;19(11):2366-74. doi: 10.1007/s10995-015-1754-6 [published Online First: 2015/07/15]
14. Friedrisch JR, Cançado RD. Intravenous ferric carboxymaltose for the treatment of iron deficiency anemia. *Revista Brasileira de Hematologia e Hemoterapia* 2015;37(6):400-05. doi: 10.1016/j.bjhh.2015.08.012
15. Pasricha SR, Tye-Din J, Muckenthaler MU, et al. Iron deficiency. *Lancet* 2020 doi: 10.1016/S0140-6736(20)32594-0 [published Online First: 2020/12/08]
16. Shand AW, Bell J, Henry A, et al. Rapid increase in intravenous iron therapy for women of reproductive age in Australia. *Med J Aust* 2020 doi: 10.5694/mja2.50618 [published Online First: 2020/05/27]

For peer review only

For peer review only - http://bmjopen.bmj.com/site/about/guidelines.xhtml

17. Qassim A, Grivell RM, Henry A, et al. Intravenous or oral iron for treating iron deficiency anaemia during pregnancy: systematic review and meta-analysis. *Med J Aust* 2019;211(8):367-73. doi: 10.5694/mja2.50308 [published Online First: 2019/08/24]
18. Achebe MM, Gafter-Gvili A. How I treat anemia in pregnancy: iron, cobalamin, and folate. *Blood* 2017;129(8):940-49. doi: 10.1182/blood-2016-08-672246 [published Online First: 2016/12/31]
19. Prevalence of low birthweight babies (% of births) - Malawi Washington DC: World Bank; 2021 [Available from: https://data.worldbank.org/indicator/SH.STA.BRTW.ZS?locations=MW accessed 18th March 2021.
20. Boudova S, Divala T, Mawindo P, et al. The prevalence of malaria at first antenatal visit in Blantyre, Malawi declined following a universal bed net campaign. *Malar J* 2015;14:422. doi: 10.1186/s12936-015-0945-3 [published Online First: 2015/10/30]
21. Haider BA, Olofin I, Wang M, et al. Anaemia, prenatal iron use, and risk of adverse pregnancy outcomes: systematic review and meta-analysis. *BMJ* 2013;346:f3443. doi: 10.1136/bmj.f3443
22. Bah A, Pasricha SR, Jallow MW, et al. Serum Hepcidin Concentrations Decline during Pregnancy and May Identify Iron Deficiency: Analysis of a Longitudinal Pregnancy Cohort in The Gambia. *J Nutr* 2017;147(6):1131-37. doi: 10.3945/jn.116.245373
23. Breymann C, Milman N, Mezzacasa A, et al. Ferric carboxymaltose vs. oral iron in the treatment of pregnant women with iron deficiency anemia: an international, open-label, randomized controlled trial (FER-ASAP). *J Perinat Med* 2017;45(4):443-53. doi: 10.1515/jpm-2016-0050 [published Online First: 2016/06/10]
24. Liang K-Y, Zeger SL. Longitudinal data analysis of continuous and discrete responses for pre-post designs. *Sankhyā: The Indian Journal of Statistics, Series B* 2000;62(1):134-48. doi: https://www.jstor.org/stable/25053123

For peer review only

Figure heading, caption and legend

Figure 1

Figure caption and legend: *REVAMP Trial schema. V0 – visit 0; V1 – Visit 1; V2 – Visit 2; V4 – Visit 4; V7 – Visit 7; V8 – Visit 8; Abbreviations: iv – Intravenous; wks – weeks.*

For peer review only

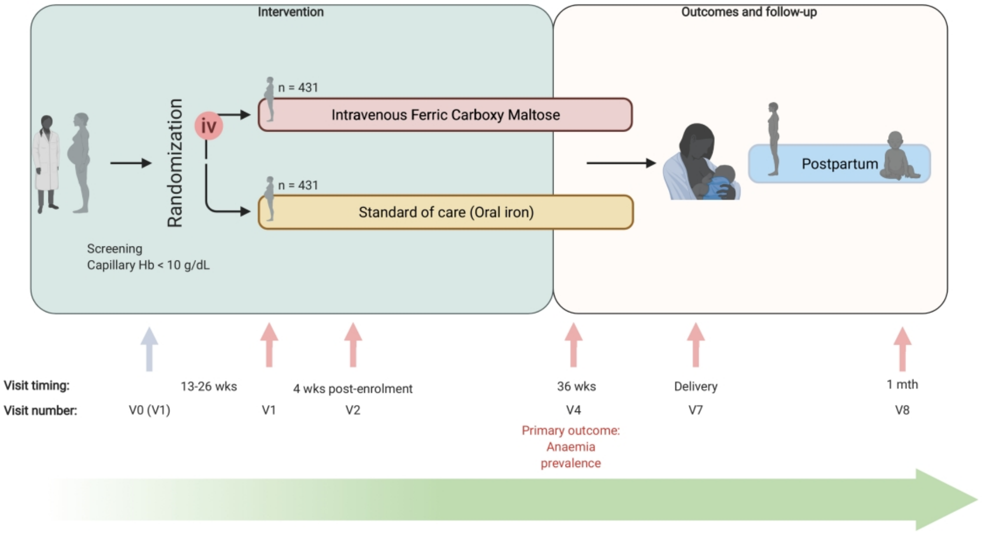

Figure 1: REVAMP Trial schema. V0 – visit 0; V1 – Visit 1; V2 – Visit 2; V4 – Visit 4; V7 – Visit 7; V8 – Visit 8; Abbreviations: iv – Intravenous; wks – weeks.

Reporting checklist for protocol of a clinical trial.

Based on the SPIRIT guidelines.

Instructions to authors

Complete this checklist by entering the page numbers from your manuscript where readers will find each of the items listed below.

Your article may not currently address all the items on the checklist. Please modify your text to include the missing information. If you are certain that an item does not apply, please write "n/a" and provide a short explanation.

Upload your completed checklist as an extra file when you submit to a journal.

In your methods section, say that you used the SPIRITreporting guidelines, and cite them as:

Chan A-W, Tetzlaff JM, Gøtzsche PC, Altman DG, Mann H, Berlin J, Dickersin K, Hróbjartsson A, Schulz KF, Parulekar WR, Krleža-Jerić K, Laupacis A, Moher D. SPIRIT 2013 Explanation and Elaboration: Guidance for protocols of clinical trials. BMJ. 2013;346:e7586

		Reporting Item	Page Number
**Administrative information**			1
Title	#1	Descriptive title identifying the study design, population, interventions, and, if applicable, trial acronym	1
Trial registration	#2a	Trial identifier and registry name. If not yet registered, name of intended registry	2 & 4
Trial registration: data set	#2b	All items from the World Health Organization Trial Registration Data Set	4
Protocol version	#3	Date and version identifier	4
Funding	#4	Sources and types of financial, material, and other support	26
Roles and responsibilities: contributorship	#5a	Names, affiliations, and roles of protocol contributors	1 & 2
Roles and responsibilities: sponsor contact information	#5b	Name and contact information for the trial sponsor	4
Roles and responsibilities:	#5c	Role of study sponsor and funders, if any, in study design; collection, management, analysis, and interpretation of data;	4

For peer review only

sponsor and funder		writing of the report; and the decision to submit the report for publication, including whether they will have ultimate authority over any of these activities	
Roles and responsibilities: committees	#5d	Composition, roles, and responsibilities of the coordinating centre, steering committee, endpoint adjudication committee, data management team, and other individuals or groups overseeing the trial, if applicable (see Item 21a for data monitoring committee)	19
**Introduction**			
Background and rationale	#6a	Description of research question and justification for undertaking the trial, including summary of relevant studies (published and unpublished) examining benefits and harms for each intervention	7
Background and rationale: choice of comparators	#6b	Explanation for choice of comparators	20
Objectives	#7	Specific objectives or hypotheses	9,10
Trial design	#8	Description of trial design including type of trial (eg, parallel group, crossover, factorial, single group), allocation ratio, and framework (eg, superiority, equivalence, non-inferiority, exploratory)	10
**Methods: Participants, interventions, and outcomes**			
Study setting	#9	Description of study settings (eg, community clinic, academic hospital) and list of countries where data will be collected. Reference to where list of study sites can be obtained	10,11
Eligibility criteria	#10	Inclusion and exclusion criteria for participants. If applicable, eligibility criteria for study centres and individuals who will perform the interventions (eg, surgeons, psychotherapists)	11,12
Interventions: description	#11a	Interventions for each group with sufficient detail to allow replication, including how and when they will be administered	12,13
Interventions: modifications	#11b	Criteria for discontinuing or modifying allocated interventions for a given trial participant (eg, drug dose change in response to harms, participant request, or improving / worsening disease)	13
Interventions: adherance	#11c	Strategies to improve adherence to intervention protocols, and any procedures for monitoring adherence (eg, drug tablet return; laboratory tests)	13

For peer review only

Interventions: concomitant care	#11d	Relevant concomitant care and interventions that are permitted or prohibited during the trial	13
Outcomes	#12	Primary, secondary, and other outcomes, including the specific measurement variable (eg, systolic blood pressure), analysis metric (eg, change from baseline, final value, time to event), method of aggregation (eg, median, proportion), and time point for each outcome. Explanation of the clinical relevance of chosen efficacy and harm outcomes is strongly recommended	21
Participant timeline	#13	Time schedule of enrolment, interventions (including any run-ins and washouts), assessments, and visits for participants. A schematic diagram is highly recommended (see Figure)	10,18, 19,20
Sample size	#14	Estimated number of participants needed to achieve study objectives and how it was determined, including clinical and statistical assumptions supporting any sample size calculations	22, 23
Recruitment	#15	Strategies for achieving adequate participant enrolment to reach target sample size	18
**Methods: Assignment of interventions (for controlled trials)**			
Allocation: sequence generation	#16a	Method of generating the allocation sequence (eg, computer-generated random numbers), and list of any factors for stratification. To reduce predictability of a random sequence, details of any planned restriction (eg, blocking) should be provided in a separate document that is unavailable to those who enrol participants or assign interventions	13
Allocation concealment mechanism	#16b	Mechanism of implementing the allocation sequence (eg, central telephone; sequentially numbered, opaque, sealed envelopes), describing any steps to conceal the sequence until interventions are assigned	13
Allocation: implementation	#16c	Who will generate the allocation sequence, who will enrol participants, and who will assign participants to interventions	13
Blinding (masking)	#17a	Who will be blinded after assignment to interventions (eg, trial participants, care providers, outcome assessors, data analysts), and how	13,14
Blinding (masking): emergency unblinding	#17b	If blinded, circumstances under which unblinding is permissible, and procedure for revealing a participant's allocated intervention during the trial	13,14

For peer review only

Methods: Data collection, management, and analysis

Data collection plan	#18a	Plans for assessment and collection of outcome, baseline, and other trial data, including any related processes to promote data quality (eg, duplicate measurements, training of assessors) and a description of study instruments (eg, questionnaires, laboratory tests) along with their reliability and validity, if known. Reference to where data collection forms can be found, if not in the protocol	20
Data collection plan: retention	#18b	Plans to promote participant retention and complete follow-up, including list of any outcome data to be collected for participants who discontinue or deviate from intervention protocols	20,21
Data management	#19	Plans for data entry, coding, security, and storage, including any related processes to promote data quality (eg, double data entry; range checks for data values). Reference to where details of data management procedures can be found, if not in the protocol	24
Statistics: outcomes	#20a	Statistical methods for analysing primary and secondary outcomes. Reference to where other details of the statistical analysis plan can be found, if not in the protocol	23
Statistics: additional analyses	#20b	Methods for any additional analyses (eg, subgroup and adjusted analyses)	n/a: statistical analysis plan to be published separately
Statistics: analysis population and missing data	#20c	Definition of analysis population relating to protocol non-adherence (eg, as randomised analysis), and any statistical methods to handle missing data (eg, multiple imputation)	23 (SAP to be published separately)
**Methods: Monitoring**			
Data monitoring: formal committee	#21a	Composition of data monitoring committee (DMC); summary of its role and reporting structure; statement of whether it is independent from the sponsor and competing interests; and reference to where further details about its charter can be found, if not in the protocol. Alternatively, an explanation of why a DMC is not needed	19
Data monitoring: interim analysis	#21b	Description of any interim analyses and stopping guidelines, including who will have access to these interim results and make the final decision to terminate the trial	23,24
Harms	#22	Plans for collecting, assessing, reporting, and managing solicited	21,22

For peer review only

		and spontaneously reported adverse events and other unintended effects of trial interventions or trial conduct	
Auditing	#23	Frequency and procedures for auditing trial conduct, if any, and whether the process will be independent from investigators and the sponsor	19
**Ethics and dissemination**			
Research ethics approval	#24	Plans for seeking research ethics committee / institutional review board (REC / IRB) approval	4
Protocol amendments	#25	Plans for communicating important protocol modifications (eg, changes to eligibility criteria, outcomes, analyses) to relevant parties (eg, investigators, REC / IRBs, trial participants, trial registries, journals, regulators)	4
Consent or assent	#26a	Who will obtain informed consent or assent from potential trial participants or authorised surrogates, and how (see Item 32)	See supplementary material 1
Consent or assent: ancillary studies	#26b	Additional consent provisions for collection and use of participant data and biological specimens in ancillary studies, if applicable	See supp. Material 1
Confidentiality	#27	How personal information about potential and enrolled participants will be collected, shared, and maintained in order to protect confidentiality before, during, and after the trial	23,24
Declaration of interests	#28	Financial and other competing interests for principal investigators for the overall trial and each study site	25
Data access	#29	Statement of who will have access to the final trial dataset, and disclosure of contractual agreements that limit such access for investigators	23,24
Ancillary and post trial care	#30	Provisions, if any, for ancillary and post-trial care, and for compensation to those who suffer harm from trial participation	21
Dissemination policy: trial results	#31a	Plans for investigators and sponsor to communicate trial results to participants, healthcare professionals, the public, and other relevant groups (eg, via publication, reporting in results databases, or other data sharing arrangements), including any publication restrictions	8,24
Dissemination policy: authorship	#31b	Authorship eligibility guidelines and any intended use of professional writers	24

For peer review only

Dissemination policy: reproducible research	#31c	Plans, if any, for granting public access to the full protocol, participant-level dataset, and statistical code	n/a; statistical analyses plan to be published separately

Appendices

Informed consent materials	#32	Model consent form and other related documentation given to participants and authorised surrogates	Supplementary material 1
Biological specimens	#33	Plans for collection, laboratory evaluation, and storage of biological specimens for genetic or molecular analysis in the current trial and for future use in ancillary studies, if applicable	14,15, 16

None The SPIRIT Explanation and Elaboration paper is distributed under the terms of the Creative Commons Attribution License CC-BY-NC. This checklist can be completed online using https://www.goodreports.org/, a tool made by the EQUATOR Network in collaboration with Penelope.ai

For peer review only

BMJ Open

Protocol for a multicentre, parallel-group, open-label randomised controlled trial comparing ferric carboxymaltose with the standard of care in anaemic Malawian pregnant women – The REVAMP trial

Journal:	*BMJ Open*
Manuscript ID	bmjopen-2021-053288.R1
Article Type:	Protocol
Date Submitted by the Author:	25-Aug-2021

Complete List of Authors:	Mwangi, Martin; University of Malawi College of Medicine, Training and Research Unit of Excellence (TRUE); University of Malawi College of Medicine, School of Public Health and Family Medicine, Department of Public Health<br>Mzembe, Glory; University of Malawi College of Medicine, Training and Research Unit of Excellence (TRUE); University of Malawi College of Medicine, School of Public Health and Family Medicine, Department of Public Health<br>Moya, Ernest; University of Malawi College of Medicine, Training and Research Unit of Excellence (TRUE); University of Malawi College of Medicine, School of Public Health and Family Medicine, Department of Public Health<br>Braat, Sabine ; University of Melbourne School of Population and Global Health, Centre for Epidemiology and Biostatistics; The University of Melbourne Melbourne Medical School, Department of Infectious Diseases<br>Harding, Rebecca; The Walter and Eliza Hall Institute of Medical Research, Population Health and Immunity Division,<br>Robberstad, Bjarne; University of Bergen, Department of Global Public Health and Primary Care<br>Simpson, Julie; University of Melbourne School of Population and Global Health, Centre for Epidemiology and Biostatistics<br>Stones, William; University of Malawi, Obstetrics & Gynaecology<br>Rogerson, Stephen; University of Melbourne, Department of Medicine at the Peter Doherty Institute for Infection and Immunity; The University of Melbourne Melbourne Medical School, Department of Infectious Diseases<br>Biselele, Kabeya; Zomba Central Hospital<br>Chinkhumba, Jobiba; University of Malawi College of Medicine, Malaria Alert Centre<br>Larson, Leila; University of South Carolina Arnold School of Public Health, Department of Health Promotion, Education, and Behavior<br>Ataíde, Ricardo; The Walter and Eliza Hall Institute of Medical Research, Population Health and Immunity Division; The University of Melbourne, Department of Infectious Diseases<br>Phiri, Kamija; University of Malawi College of Medicine, Training and Research Unit of Excellence (TRUE); University of Malawi College of Medicine, School of Public Health and Family Medicine, Department of Public Health<br>Pasricha, Sant-Rayn; Walter and Eliza Hall Institute of Medical Research,

	Population Health and Immunity Division; The Royal Melbourne Hospital, Diagnostic Haematology and Clinical Haematology, The Peter MacCallum Cancer Centre
**Primary Subject Heading**:	Haematology (incl blood transfusion)
Secondary Subject Heading:	Global health, Haematology (incl blood transfusion), Nutrition and metabolism, Public health, Infectious diseases
Keywords:	Anaemia < HAEMATOLOGY, Nutrition < TROPICAL MEDICINE, Epidemiology < TROPICAL MEDICINE, Clinical trials < THERAPEUTICS, PUBLIC HEALTH, Epidemiology < INFECTIOUS DISEASES

I, the Submitting Author has the right to grant and does grant on behalf of all authors of the Work (as defined in the below author licence), an exclusive licence and/or a non-exclusive licence for contributions from authors who are: i) UK Crown employees; ii) where BMJ has agreed a CC-BY licence shall apply, and/or iii) in accordance with the terms applicable for US Federal Government officers or employees acting as part of their official duties; on a worldwide, perpetual, irrevocable, royalty-free basis to BMJ Publishing Group Ltd ("BMJ") its licensees and where the relevant Journal is co-owned by BMJ to the co-owners of the Journal, to publish the Work in this journal and any other BMJ products and to exploit all rights, as set out in our licence.

The Submitting Author accepts and understands that any supply made under these terms is made by BMJ to the Submitting Author unless you are acting as an employee on behalf of your employer or a postgraduate student of an affiliated institution which is paying any applicable article publishing charge ("APC") for Open Access articles. Where the Submitting Author wishes to make the Work available on an Open Access basis (and intends to pay the relevant APC), the terms of reuse of such Open Access shall be governed by a Creative Commons licence – details of these licences and which Creative Commons licence will apply to this Work are set out in our licence referred to above.

Other than as permitted in any relevant BMJ Author's Self Archiving Policies, I confirm this Work has not been accepted for publication elsewhere, is not being considered for publication elsewhere and does not duplicate material already published. I confirm all authors consent to publication of this Work and authorise the granting of this licence.

For peer review only

Protocol for a multicentre, parallel-group, open-label randomised controlled trial comparing ferric carboxymaltose with the standard of care in anaemic Malawian pregnant women – The REVAMP trial

Authors

Martin N Mwangi[1,2], Glory Mzembe[1,2], Ernest Moya[1,2], Sabine Braat[3,4,5], Rebecca Harding[3], Bjarne Robberstad[6], Julie Simpson[5], William Stones[2], Stephen Rogerson,[4,7] Kabeya Biselele[8], Jobiba Chinkhumba[9], Leila Larson[10], Ricardo Ataíde[3,4], Kamija S Phiri[1,2], Sant-Rayn Pasricha[3,11,12]

Institutions

1. Training and Research Unit of Excellence (TRUE), Mandala, No. 1 Kufa Road, Chichiri, BT3, Blantyre, Malawi.
2. School of Public Health and Family Medicine, Department of Public Health, Kamuzu University of Health Sciences (KUHeS), Chichiri, BT3, Blantyre, Malawi.
3. Population Health and Immunity Division, The Walter and Eliza Hall Institute of Medical Research, 1G Royal Parade, Parkville, Melbourne 3052, Australia.
4. Department of Infectious Diseases, Melbourne Medical School, The University of Melbourne, Melbourne, Australia.
5. Centre for Epidemiology and Biostatistics, Melbourne School of Population and Global Health, The University of Melbourne, Melbourne, Australia.
6. Department of Global Public Health and Primary Care, University of Bergen, Bergen, Norway.
7. Department of Medicine at the Peter Doherty Institute for Infection and Immunity, University of Melbourne, Melbourne, Australia.
8. Zomba Central Hospital, Department of Obstetrics and Gynecology, Zomba, Malawi.

For peer review only

9. Malaria Alert Centre, Kamuzu University of Health Sciences (KUHeS), Chichiri, BT3, Blantyre, Malawi.
10. Department of Health Promotion, Education, and Behaviour, Arnold School of Public Health, University of South Carolina, Columbia, South Carolina, USA.
11. Diagnostic Haematology, The Royal Melbourne Hospital; and Clinical Haematology, The Peter MacCallum Cancer Centre and The Royal Melbourne Hospital, Parkville VIC 3050 Australia.
12. Department of Medical Biology, Faculty of Medicine, Dentistry and Health Sciences, The University of Melbourne, Melbourne, Australia.

Protocol registry number: ACTRN12618001268235

Corresponding author:

Martin N. Mwangi, P.O. Box 30538, Chichiri, BT3, Blantyre, Malawi. Email: mmwangi@true.mw

Word count: 4,962 words. (Abstract) 299 words. *(excluding title page, abstract, references, figures and tables)*

For peer review only

ABSTRACT

Introduction: Anaemia in pregnancy remains a critical global health problem, affecting 46% of pregnant women in Africa and 49% in Asia. Oral iron therapy requires extended adherence to achieve correction of anaemia and replenishment of iron stores. Ferric carboxymaltose (FCM) is a recently established intravenous iron formulation associated with substantial advantages in safety, speed of delivery and total dose deliverable in a single infusion. We aim to determine whether FCM given once during the second trimester of pregnancy compared to standard oral iron distributed through routine antenatal services is effective and safe for treatment of moderate to severe maternal anaemia in sub-Saharan Africa.

Methods and analysis: REVAMP is a two-arm confirmatory individually-randomised controlled trial set in Blantyre and Zomba districts in Malawi. The trial will randomise 862 women in the second trimester of pregnancy with a capillary haemoglobin concentration below 10.0g/dL. The study comprises two arms: (a) intravenous ferric carboxymaltose (20mg/kg up to 1000mg) given once at randomisation, and (b) standard of care oral iron (65mg elemental iron twice daily) for 90 days (or the duration of pregnancy, whichever is shorter) provided according to local health care practices. Both arms receive sulfadoxine-pyrimethamine (SP) as Intermittent Preventive Treatment in pregnancy (IPTp). The primary outcome is the prevalence of anaemia (Hb<11.0g/dL) at 36 weeks' gestation. Secondary outcomes include birth weight, gestation duration, and safety outcomes, including clinical malaria, serious perinatal events and postpartum haematologic and health-related outcomes in the mother and child.

Ethics and dissemination: Ethical approval was granted by the Research Ethics Committee (COMREC P.02/18/2357) in Malawi and the Human Research Ethics Committee (WEHI: 18/02), Melbourne, Australia. The protocol is registered with the Australian and New Zealand Clinical Trials Registry

For peer review only - http://bmjopen.bmj.com/site/about/guidelines.xhtml

(ACTRN12618001268235). The results will be shared with the local community that enabled the research, and also to the international fora.

Keywords: Anaemia, Iron Deficiency, Intravenous Iron, ferric carboxymaltose (FCM), Pregnancy, Malawi, randomised controlled trial

Data Statement

All relevant data are within the paper and its supporting information files.

Protocol version

REVAMP Trial protocol version 5.0 dated 21 November 2020

Ethics approval

College of Medicine Research Ethics Committee (COMREC P.02/18/2357) in Malawi and the Walter and Eliza Hall Institute of Medical Research Human Research Ethics Committee (WEHI: 18/02), Melbourne, Australia.

Trial Sponsor

Director - Research Support Centre.

College of Medicine, Kamuzu University of Health Sciences (KUHeS), Private Bag 360, Chichiri, Blantyre 3, Malawi.

Email: rscdirector@medcol.mw

Roles and responsibilities of sponsor and funder:

The sponsor and funder had no role in study design. They will have no role nor ultimate authority in the collection, management, analysis, and interpretation of data; writing of the report; and the decision to submit the report for publication.

For peer review only

ARTICLE SUMMARY

Strengths and limitations of this study

1. Trial setting: The trial is conducted in a low-income sub-Saharan African setting where there is a high prevalence of anaemia in pregnancy, low birth weight and *P. falciparum* malaria. The trial will provide critical new data on the effectiveness and safety of intravenous iron in a context where a) anaemia and iron deficiency rates are high, b) malaria is prevalent and causes anaemia, and c) iron interventions may increase malaria infection rates. In these settings, needs for anaemia control are critical.
2. Participant screening: Eligibility for inclusion is determined by moderate or severe anaemia detected via capillary haemoglobin estimation, which at present is a convenient and available method for assessing anaemia status that could be deployed in the high-throughput health centre setting; our results will thus provide direct information on the impact of this highly feasible screening and treatment approach for antenatal anaemia.
3. Use of ferric carboxymaltose: this is the first trial in a low-income field setting to use a recently established intravenous iron formulation for the treatment of anaemia in pregnancy; previous trials in low-income settings have used older formulations such as iron sucrose and iron dextrans, which may have a higher risk of adverse events and take longer to infuse, and thus are suboptimal for routine deployment in the health centre setting.
4. Outcomes: our trial includes a critical primary outcome of anaemia before delivery, as well as key secondary outcomes to assess the benefit of the trial intervention for mother and neonate, and detailed safety assessments using active and passive data collection.
5. Sample size: this is the largest trial of ferric carboxymaltose in pregnancy. It is also among the largest trials of ferric carboxymaltose (and intravenous iron) ever conducted. The study will thus provide critical effectiveness and safety information on this drug's role in pregnancy globally.

For peer review only - http://bmjopen.bmj.com/site/about/guidelines.xhtml

INTRODUCTION

Anaemia during pregnancy remains a critical global health problem. Almost 40% of pregnant women worldwide are anaemic, including 46% of pregnant women in Africa and 49% in Asia.[1] Anaemia in pregnancy is very common and mostly results from iron deficiency and is associated with critical risks for both mother (e.g. life-threatening complications of postpartum haemorrhage) and child (especially prematurity and low birth weight,[2] which are associated with increased risk of neonatal and infant mortality,[3] and reduced iron stores in infancy with increased risk of subsequent anaemia and impaired development).[4] Severe anaemia in pregnancy is associated with a significantly increased risk of maternal mortality.[5] Worldwide, 15%-20% of births (>20 million annually, including 13% in sub-Saharan Africa) are low birthweight. At the same time, each year, over 1 million children die from preterm birth complications, making prevention and treatment of antenatal anaemia a crucial component of efforts to reduce low birth weight.

Systematic reviews confirm likely benefits from iron on maternal outcomes, including anaemia (70% reduction) and trends towards favourable infant outcomes, including increased birth weight and extended gestation duration.[6] A trial of oral iron compared with placebo in Kenyan pregnant women confirmed a benefit from iron on birth weight and gestation duration.[7] Global recommendations for managing anaemia in pregnancy in lower and middle income countries (LMICs) currently advise that women be treated with high-dose daily oral iron (120 mg of elemental iron) supplementation for three months.[8 9] However, oral iron is often poorly tolerated due to gastrointestinal adverse effects,[10] limiting adherence; lower doses with intermitted dosing have been used in non-pregnant women.[11] Delivery of iron during pregnancy requires high-quality performance of the primary health system. In Malawi, 10.6% of women were reported to take no iron during pregnancy, and 37.1% take less than 60 doses, with only 33.4% taking the recommended 90 or more doses.[12] Even when delivered, oral iron frequently fails to correct anaemia. For example, 61% of Gambian pregnant women who received iron at week 20 of gestation – when their Hb was lower than 10.0g/dL - still had a Hb<10.0g/dL at 30

For peer review only

weeks' gestation.[13] Finally, women may only present for their first antenatal visit late in the second trimester, curtailing the time available to optimise iron stores with oral iron to improve foetal development and risks associated with delivery; for example, in Malawi, fewer than 30% of pregnant women attend before the sixteenth week of gestation.[14]

Over the past decade, there have been dramatic improvements in parenteral (intravenous) iron therapeutics. Ferric carboxymaltose (FCM) is a widely used formulation that can be administered in a short period (15 min) and at large doses (up to 1000 mg) in a single infusion.[15 16] FCM has revolutionised the treatment of iron-deficiency anaemia in high-income country settings and is widely used in outpatient settings, emergency departments, and primary care.[17] In pregnancy, intravenous iron has been reported to be superior to oral iron in improving haemoglobin concentrations and may even increase birth weight.[18] Intravenous iron is increasingly recommended as a suitable first-line option for women with moderate or severe iron-deficiency anaemia beyond the first trimester of pregnancy.[19] Studies have evaluated the role of older forms of intravenous iron in pregnancy in low-income settings such as India,[20 21] whilst other studies have demonstrated the feasibility of using FCM in the post-partum period in sub-Saharan Africa[22] but modern formulations capable of delivering a rapid total-dose infusion have not yet been studied in women in pregnancy in low income countries.

For peer review only

It remains challenging to directly measure iron status in the field, as biomarkers generally require analysis using centralised laboratory infrastructure. In contrast, point-of-care measurement of capillary haemoglobin is a feasible field-friendly testing strategy that can be deployed with existing technology, of which HemoCue point-of-care devices are a good example. Screen-and-treat approaches for anaemia in pregnancy must rely on haemoglobin measurement until point-of-care assays for iron status become more widely available.

FCM presents a practical opportunity for rapid correction of moderate and severe antenatal anaemia

For peer review only - http://bmjopen.bmj.com/site/about/guidelines.xhtml

in a single dose, potentially improving maternal and neonatal clinical outcomes for pregnant women. It is, therefore, urgent to test this therapy in a low-income field setting where the burden of anaemia-related disease is heaviest. In Malawian pregnant women with moderate or severe anaemia during the second trimester of pregnancy, we aim to evaluate whether a single dose of intravenous ferric carboxymaltose is superior to oral iron provided via standard-of-care approaches.

METHODS AND ANALYSIS

Patient and Public Involvement

We held discussions with national policy stakeholders during the study planning stage, including the Ministry of Health. We discussed how the research might align with national research and health service priorities. Local community engagement was done via public meetings; the potential participants were first involved in the study's design during these meetings. We discussed the study with the traditional leaders, including traditional authorities (TAs), group village headmen (GVH), the village development committee (VDC), and ward councillors (political figures). The GVH were requested to cascade the information to the village chiefs who took the information to the community. Health workers were accessed via the District Health Offices in Blantyre and Zomba districts. Health workers from the participating health facilities, including the health surveillance assistants (HSAs) who directly work with the community, were informed about the proposed research and discussions on priorities beneficial to the community were discussed. The public, potential participants, and health workers identified malaria and anaemia as major public health issues in the community. Women outlined their experiences with iron supplements during their past pregnancies and identified the development of tolerable iron formulations as a research priority. Dissemination of findings at the national and community level will follow the schema used during study design and inception as outlined above.

For peer review only - http://bmjopen.bmj.com/site/about/guidelines.xhtml

Trial objectives

The primary objective of the trial is to determine whether, in Malawian women in the second trimester of pregnancy with moderate or severe anaemia, a single dose of FCM up to 1000mg is superior to standard-of-care (i.e. oral iron provided through local health services), in reducing maternal anaemia before delivery (at 36 weeks' gestation).

The secondary objectives are:

During pregnancy and up to one month postpartum, to determine the effects of FCM (compared with standard-of-care) on:

Effectiveness:

- Maternal haemoglobin concentration and iron status (measured through iron biomarkers), and
- Critical neonatal outcomes including birth weight (low birth weight), gestation duration (prematurity), small for gestational age and other perinatal outcomes.

Safety:

- Maternal and neonatal adverse events, including infection episodes, serious maternal complications, and hypophosphatemia.

Exploratory objectives up to one month postpartum for subsequent hypothesis generation are related to maternal cognition, depression, and fatigue, as well as costs of health care.

Study design

REVAMP is a multicentre, open-label, two-arm, parallel-group individually randomised controlled trial (RCT). An open-label (rather than blinded) design was selected as it was considered unfeasible to deliver the placebo intravenous infusions to Malawian pregnant women in this field-trial context (the

For peer review only

drug is dark-coloured). The trial recruited pregnant women in their second trimester and is following them until one month postpartum, after which we will report on the primary and secondary objectives. Extended follow-up of mothers and infants to 12-months postpartum is planned, as is a range of exploratory economic, biological and clinical outcomes. These analyses are beyond the scope of this protocol, as are the exploratory outcomes collected up to one month postpartum, and they will be reported separately. The trial design is summarised in **Figure 1**.

Study settings and participants

In Malawi, the average gestation age at the first antenatal clinic (ANC) visit is 22 weeks, with 61% presenting before 24 weeks. The 2015-16 DHS survey estimated a prevalence of anaemia (Hb < 11.0g/dL) in pregnant women in Malawi at 45.1%, including 22.4% of women with moderate or severe anaemia.[12] The prevalence of low birth weight in Malawi was around 14% in 2015.[23] Malawi is endemic for malaria and the prevalence of *Plasmodium spp.* parasitaemia at the first ANC visit in the study site exceeds 15%.[24] About 87% of women consume at least one dose of intermittent preventive treatment during pregnancy (IPTp), but this falls to 30% for women taking three or more doses.[12]

The REVAMP study has two major research sites namely Limbe Health Centre and Zomba Central Hospital located in Blantyre and Zomba districts respectively, in Southern Malawi. The two major sites act as the study coordination centres in the respective districts. The Blantyre site serves as the base for five urban health centres/antenatal clinics, whilst the Zomba site serves as the base for nine (four urban and five rural) health centres/antenatal clinics. Women are screened at the antenatal clinics and referred to the coordinating research site for enrolment, treatment and follow-up visits. Deliveries occur in government-operated birth suites attached to the study site with referral to a district hospital where required for obstetric indications.

For peer review only - http://bmjopen.bmj.com/site/about/guidelines.xhtml

Eligibility criteria

Women are only randomised if they fulfil all inclusion criteria and none of the exclusion criteria.

Inclusion criteria:

1. Confirmed singleton pregnancy at 13-26 weeks' gestation
2. Moderate to severe anaemia (capillary haemoglobin <10.0g/dL measured by HemoCue 301+) not clinically deemed to require an immediate blood transfusion. We excluded women with mild anaemia as thresholds to distinguish mild anaemia from health are indistinct, and because moderate and severe anaemia have an increased link to adverse maternal and child health outcomes.[25]
3. Negative malaria parasitaemia at screening and no positive malaria rapid diagnostic test within the previous seven days
4. Resident in the study catchment area of Blantyre and Zomba district
5. Plan to deliver at a health facility
6. Written informed consent, including assent if <18 years old (consent of a parent or guardian plus the participant's assent).

Exclusion criteria:

1. Hypersensitivity to any of the study drugs
2. Clinical symptoms of malaria or positive malaria rapid diagnostic test within the previous seven days, or symptoms of bacterial infection, at screening
3. Any condition requiring hospitalisation or serious concomitant illness
4. Chronic illness that may adversely affect foetal growth and viability
5. Severe anaemia clinically judged as requiring a blood transfusion (usually Hb <5.0g/dL)
6. Pre-eclampsia at screening (new onset of hypertension, proteinuria and swelling in the legs, feet, and hands).

For peer review only

Trial Interventions

Participants are randomly assigned to receive one of the following interventions:

1. **Intravenous iron**: ferric carboxymaltose 20mg/kg up to 1000mg, diluted in normal saline and given over 15 minutes, once after randomisation. The study clinician administers the drug, and women remain under observation for 45 minutes after drug administration. Women receiving FCM are treated in rooms equipped with a trolley containing adrenaline, hydrocortisone, intravenous fluids and antihistamines, airway equipment and an oxygen concentrator. Staff are trained in the management of allergic reactions. Ferric carboxymaltose, manufactured by Vifor Pharma, was purchased at full-price from Aspen Pharma in Australia and shipped to Malawi.
2. **Standard-of-care - Oral iron treatment course**: oral iron- 200mg ferrous sulphate (approx. 65mg elemental iron) twice daily for 90 days or the remaining duration of pregnancy, whichever is shorter. Oral iron is provided by health workers using the same education messaging and similar packaging as provided by the routine health system to reflect real-life health service conditions.

Following national guidelines, both groups receive IPTp with 1500 mg Sulfadoxine and 75 mg Pyrimethamine (SP), as three tablets of SP of fixed dose (500mg/25mg) at least three times during pregnancy, at least four weeks apart. The IPTp-SP course at four weeks' post-randomisation is directly observed by study staff. HIV positive women in Malawi are not offered SP because they are already on Cotrimoxazole prophylaxis. The baseline dose is also directly administered by study staff if the participant did not receive it through the health service. All participants also receive an insecticide-treated bed net.

Randomisation, allocation concealment, and blinding

Participants are randomly allocated to one of the two treatments arms with 1:1 allocation via a computer-generated randomisation schedule of randomly permuted blocks stratified by research site

For peer review only - http://bmjopen.bmj.com/site/about/guidelines.xhtml

(Blantyre, Zomba) to achieve a balance between the arms within each site. The randomisation list was generated by an independent statistician at the University of Melbourne (Australia).

Individual participant codes were pre-packed in sealed envelopes by an independent researcher not associated with the study and held securely at the research sites. Eligible participants who meet all inclusion/exclusion criteria are sequentially allocated participant identification numbers within the research site, and their allocation to study group is revealed after opening the corresponding sealed envelope. Although the trial is open-label, laboratory scientists measuring haemoglobin concentration, midwives collecting birth outcome data, and investigators and researchers in Australia (including data managers and statisticians in Melbourne) are blinded to the treatment allocation during the conduct of the trial until the database is locked and ready for unblinding.

Recruitment and Visits

Table 1 shows the schedule of activities per visit for the mother, and **Table 2** shows the schedule of activities per visit for the neonate.

For peer review only - http://bmjopen.bmj.com/site/about/guidelines.xhtml

Table 1: Planned activities per visit for mothers

Protocol activity	Visit 0	Visit 1	Visit 2	Visit 3[j]	Visit 4	Visit 5 – 6[j]	Visit 7	Visit 8
	Day -7 to 0	Day 0	Day 28 ±2 days	34 wks. gestation ±2 days	36 wks. gestation ±2 days	38-40 wks. gestation ± 2 days	Delivery + 1 day	Day 28 postpartum ±2 days

For peer review only

Location of visit	*Antenatal Clinic*	*Research Site[a]*	*Research Site[a]*	*Home[b]*	*Research Site[a]*	*Home[b]*	*Research Site[a]*	*Research Site[a]*
Informed consent process for pre-screening	X							
Pre-screening	X							
Detailed Informed consent process	X	X						
Screening		X						
Medical & obstetric history		X						
Demographics		X						
Maternal physical examination[d]		X [h]	X [i]		X [i]		X [h]	X [i]
Ultrasound scan **(Fetal Biometry)[d]**		X	X		X			
Randomise participant		X						
Administer treatment		X						
Intermittent preventive treatment[c]		X	X		X			
Laboratory procedures								
Capillary haemoglobin[d]	X	X	X	X	X	X	X	X
Full blood count[d]		X	X		X		X	X
Malaria diagnostics[d, e]	X	X	X		X		X	X
Serum for iron markers tests[f]		X	X		X		X	X

For peer review only

Serum for inflammatory markers tests[g]		X	X		X		X	X
Phosphate		X	X		X		X	X
Adverse events[d]		X	X		X		X	X
Morbidities								X
Missed visits[d]			X	X	X	X	X	X
End of study			X	X	X	X	X	X

[a] Visit conducted at the research site/health facility; [b] Visit conducted at the participant's home; [c] Assessing if intermittent preventive treatments sulphadoxine pyrimethamine and albendazole were given; [d] Protocol activities are also collected at any unscheduled visits; [e] Malaria diagnostics include malaria rapid diagnostic test, malaria microscopy, [f] Serum for iron markers tests include serum ferritin; [g] Serum for inflammatory markers tests include C-reactive protein and alpha-1 glycoprotein; [h] Complete maternal physical examination includes general appearance, throat, neck, thyroid, musculoskeletal, skin, lymph nodes, extremities, pulses, pulmonary, cardiac, abdominal, and neurological examination; [i] Limited maternal physical examination includes general appearance, brief pulmonary, cardiac, abdominal and neurological examination. [j] Since the opening of the trial, visits 3, 5 and 6 have been suspended since April 2020 due to the risk of exposing communities to COVID-19 during home visits.

For peer review only

Table 2: Details of planned activities per visit for neonates

Protocol activity	Visit 7	Visit 8
	Delivery +1 day	Day 28 Postpartum ±2 days
Location of visit	*Research Site[a]*	*Research Site[a]*
Pregnancy outcome	X	
Physical examination and anthropometry [b, c]	X[f]	X[g]
Laboratory procedures		
Capillary haemoglobin	X[h]	
Full blood count[b]	X[h]	X
Malaria diagnostics[d]	X[h]	X
Serum for iron markers tests[e]	X[h]	X
Vaccination and Vit A supplementation status		X
Adverse events[b]	X	X
Morbidities		X
Missed visits[b]		X
End of study	X	X

[a] Visit done at the research site/ health facility; [b] Protocol activities are also collected at any unscheduled visits; [c] Anthropometry includes assess the infant's weight, length, and head circumference; [d] Malaria diagnostics include malaria rapid diagnostic test, malaria microscopy, malaria filter paper for polymerase chain reaction and histology at delivery; [e] Serum for iron markers tests include serum ferritin; [f] The complete physical examination includes general appearance, throat, neck, thyroid, musculoskeletal, skin, lymph nodes, extremities, pulses, pulmonary, cardiac, abdominal, and neurological examination [g] The limited examination includes general appearance, brief pulmonary, cardiac, abdominal and neurological examination; [h] These assessments are collected via cord blood.

For peer review only

Screening and enrolment of participants

Study recruitment opened on November 12, 2018; the final participant was recruited on March 02, 2021. Study activities are detailed in Tables 1 and 2.

Pre-screening and screening, visit 0 (day -7 to 0)

Women attending their first antenatal visit at ANCs are given general information (aims and procedures) about the study by a trained research nurse and invited to consent to pre-screening procedures. As the ANC nurse cares for attendees, the research nurse pre-screens potential participants by noting apparent gestation age based on clinical examination and last menstrual period and then tests for anaemia with capillary Hb (HemoCue 301+, Angelholm Sweden) and *Plasmodium* parasitaemia by rapid diagnostic test (SD Bioline Malaria AG P.F/PAN, Standard Diagnostics, Inc.). Potentially eligible participants receive further information about the study. Willing participants are referred to the research site for additional evaluation, full consenting procedure and recruitment.

Enrolment, visit 1 (day 0)

As presented in Table 1, at the health facility, potentially eligible participants undergo ultrasound scanning (within two days from the initial pre-screening visit) to confirm singleton pregnancy and determine gestation age (i.e. crown-rump length for less than or equal to 16 weeks, femur length and head and abdominal circumference for more than 16 weeks). Written informed consent is then obtained for participation in the trial. For consenting participants, a physical examination is conducted, and baseline data are collected, comprising demographic information and medical and obstetric history. Following the collection of baseline clinical data, samples of both capillary and venous blood are collected and then participants are randomised. For women randomised to the ferric carboxymaltose arm of the trial, an intravenous cannula is inserted under aseptic precautions, and a trained nurse administers the study drug according to standard procedures and under the supervision of the study clinician. Women randomised to the standard-of-care arm of the trial are provided with

For peer review only - http://bmjopen.bmj.com/site/about/guidelines.xhtml

a full course of iron tablets, together with information delivered according to a standardised script reflecting ANC practice.

Subsequent study visits

Visit 2 (28 (±2) days after enrolment into the study): Participants receive a physical examination and an ultrasound scan before being asked for a venous blood sample (Table 1). A capillary sample is collected for assessment of *Plasmodium* parasitaemia. The participants are asked to continue their regular ongoing ANC visits through their local health centre.

Visit 3; visits 5-6 (34 weeks' gestation ±2 days; 38-40 weeks' gestation ±2 days): Participants are visited in their home at 34 weeks' gestation, and every two weeks from 38 weeks' gestation until delivery. A research nurse measures capillary Hb. Where the participant belongs to the standard-of-care trial-arm, the research nurse attempts to assess adherence. Notably, since the opening of the trial, fieldwork has been affected by the COVID-19 pandemic. Because of the risks of study workers introducing COVID-19 into remote villages, home visits were removed from the trial protocol after April 2020.

Visit 4 (36 weeks' gestation/pre-delivery ±2 days): Procedures are similar to those for Visit 2 (28-days post-treatment). This visit includes the assessment of venous haemoglobin for the primary outcome.

Visit 7 (Delivery +1 day): The study provides 24-hour cover of the study research sites' delivery suites. All participants are asked to return to the research site for delivery (unless a high-risk pregnancy requires delivery at the tertiary referral hospital). Standardised delayed cord clamping procedures are instituted. Participants delivering at home or at other health facilities are encouraged to attend the research site for assessment within 24 hours. Venous blood samples are collected during labour. Apgar scores are recorded immediately after delivery, and the newborn undergoes a full physical examination, including measurement of birth weight, length, head circumference, and assessment for congenital malformations. The type of birth and occurrence of perinatal complications (including

For peer review only

haemorrhage or need for blood transfusion) are recorded. Placental blood is drawn, and a sample of placental tissue is collected and stored in buffered formalin for subsequent histological evaluation.

Visit 8 (28 days postpartum ±2 days): Participants return to the research site together with their infants for a detailed medical examination of both mother and infant and collection of blood samples.

Unscheduled visit: Participants are asked to attend the research site when symptomatically unwell. They are managed according to national standard treatment guidelines by a trained health care provider. Blood samples for malaria RDT (and microscopy if RDT positive) and blood culture are taken if clinically indicated. The clinical diagnosis of the unscheduled visit is recorded in the participant's record. Where a participant attends another health facility or antenatal clinic, the research team extracts the missed unscheduled visit notes from the participants health book commonly known as a health "passport" in Malawi. Usually, clinical investigations and medications are indicated in the health book and can be extracted by the research team during the next scheduled visit.

Laboratory Procedures

Venous blood is measured for haemoglobin concentration using an automated analyser (Sysmex, XP 300 series, Sysmex Corporation, Kobe, Japan), for which daily two level controls are run and recorded. Serum is separated by centrifugation and stored at -80 degrees Celsius. Samples will be batched and assayed for ferritin, C-reactive protein, and phosphate in Meander Medical Centre laboratory, accreditation number M040, EN ISO 15189:2012 (Amersfoort, The Netherlands).

Data Monitoring Committee (DMC)

An independent DMC has been set up to review on a regular basis, safety and efficacy data of the ongoing trial. The DMC is comprised of international experts in clinical trials, obstetrics, epidemiology and statistics. In the advent of clear evidence of effectiveness or harm, the DMC will recommend to the sponsor and investigators whether to continue, modify, or terminate the trial on ethical grounds.

For peer review only

For peer review only - http://bmjopen.bmj.com/site/about/guidelines.xhtml

Outcomes

The primary outcome is the prevalence of maternal anaemia before delivery, defined as a venous haemoglobin concentration less than 11.0g/dL at 36 weeks' gestation. This outcome evaluates the performance of the study intervention in helping women reach labour with optimal tissue oxygenation and resilience.

Secondary outcomes assessing effectiveness in the mother are anaemia at four weeks' post-randomisation and one month postpartum, moderate/ severe anaemia, haemoglobin concentration, iron deficiency (ferritin<15mg/L adjusted for C-reactive protein), iron deficiency anaemia, and serum ferritin concentration all at four weeks post-randomisation, 36 weeks' gestation, and one month postpartum.

Effectiveness in the neonate is assessed using the outcome of mean birthweight (measured in grams within 24 hours of birth) and the secondary outcomes low birth weight (birth weight less than 2500g), gestation duration (weeks), birth length, small for gestation age, fetal loss (pregnancy loss before 28 completed weeks' gestation), stillbirth (birth of a child showing no signs of life after 28 weeks' gestation), preterm births (neonate born before 37 completed weeks of gestation), haemoglobin concentration at 1-month postpartum and infant growth (weight for age, length for age, weight for length) at one month postpartum.

Outcomes assessing safety in the mother are (serious) adverse events related to administration of FCM (events occurring during the enrolment visit), (serious) adverse events (within 14 days of randomisation, antenatal and postpartum reported separately), all-cause sick visits to the clinic (antenatal and postpartum reported separately), clinical malaria-specific visits to the clinic (antenatal and postpartum reported separately)*P. falciparum* parasitaemia (at four weeks' post-randomisation and 36 weeks' gestation), hypophosphatemia (biochemical) (at four weeks' post-randomisation and 36 weeks' gestation), inflammation (elevated C-reactive protein) (at four weeks' post-randomisation

For peer review only - http://bmjopen.bmj.com/site/about/guidelines.xhtml

and 36 weeks' gestation), and severe medical events including haemorrhage, receipt of blood transfusion, ICU admission, or mortality.

Clinical infections will be reported during unplanned visits. Clinical malaria will be defined clinically, in women who present with fever and a positive malaria test. Diarrhoea will be defined in women with more than three loose stools per day. Other clinical diagnoses will be made according to local health manuals.

Safety in the infant is assessed using (serious) adverse events, all-cause visits to the clinic, infection-related visits to the clinic, diarrhoea-related visits to the clinic, and clinical malaria-specific visits to the clinic.

In addition to the primary and secondary clinical outcomes listed above, which will support the reporting of the confirmatory RCT, data for many exploratory outcomes are being collected between enrolment and 12-months postpartum. These include assessing maternal cognition, mother-infant bonding, maternal depression and fatigue, infant neurodevelopment, infant diet and health economic data, including direct and indirect costs of health care. Also, women have given their consent for the collection of samples for future translational studies, including evaluation of the vaginal and faecal microbiome, maternal immune profiles, and effects of interventions directed towards *P. falciparum* biology.

For peer review only

Detection and reporting of Adverse Events and Serious Adverse Events

Non-serious Adverse Events (AEs) and serious adverse events (SAEs) are collected from the time consent is given until the participant completes the study (the final visit or withdrawal). These are detected either through spontaneous reports by the participant, unplanned visits to the research site or any of the participating health centres, observation by the study staff, and through standard

questioning at each visit. All SAEs are reported to the sponsor and the appropriate ethics committee, whether or not considered causally related to the study drugs.

Sample size

The sample size calculation is based on the primary maternal outcome of the proportion of women with pre-delivery anaemia (defined as venous Hb<11.0g/dL at 36 weeks' gestation). A systematic review and meta-analysis identified a 50% reduction in anaemia prevalence in women receiving oral iron.[2] In a cohort of pregnant women in the Gambia, we observed that 60% of pregnant women with Hb<10.0g/dL at 20 weeks' gestation who then receive iron interventions continue to have Hb<10.0g/dL by 30 weeks.[13] The pivotal Fer-ASAP trial (which compared FCM to oral iron in women with iron deficiency anaemia in high-income settings) demonstrated a 14% reduction in absolute anaemia prevalence compared with oral iron.[26] We hypothesise that routine iron supplementation will reduce anaemia prevalence from 100% to 60% (as seen in the Gambia,[13] and similar to data from Haider and colleagues[2]). We hypothesise that ferric carboxymaltose will result in a 10% absolute improvement in anaemia cure (to a prevalence of 50%). A total sample size of 862 pregnant women or 431 per group will provide 80% power to detect this difference, allowing for a 10% loss to follow-up and assuming a two-sided alpha of 5%. The sample size also has at least 80% power to detect an absolute difference between standard-of-care oral iron and ferric carboxymaltose of 100g in the neonatal outcome of birth weight (assuming a standard deviation of 450g and a two-sided alpha of 5%), similar to the effect size seen in a trial of Kenyan women receiving oral iron when compared to placebo.[7]

STATISTICAL ANALYSIS PLAN

A detailed statistical analysis plan will be finalised before unblinding of the database. Analyses will be performed where participants are classified according to their randomised intervention arm (i.e.

For peer review only - http://bmjopen.bmj.com/site/about/guidelines.xhtml

intention to treat principle). An available case analysis will be performed for repeated time point outcomes (e.g. anaemia) and a complete-case analysis for single time point outcomes (e.g. birth weight). Anaemia will be analysed using a log-binomial regression model, including study participants as a random intercept to account for the multiple time points. The model will include the standard-of-care (oral iron) group as the reference group. The primary maternal hypothesis will be evaluated by obtaining the estimate of the prevalence ratio of IV iron versus standard-of-care (oral iron), 95% confidence interval extracted at 36 weeks' gestation, and p-value. Birthweight will be analysed by fitting a linear regression model. The primary neonatal hypothesis will be evaluated by estimating the absolute difference in mean birth weight between IV iron and standard-of-care (oral iron) along with a corresponding 95% confidence interval and p-value. Secondary repeated time point binary outcomes will be analysed similarly to anaemia, and secondary single time point continuous outcomes will be analysed similarly to birthweight. Secondary, single time point binary outcomes (e.g., sub-optimal pregnancy outcomes) will be analysed using a log-binomial regression model and secondary, multiple time point continuous outcomes (e.g., maternal haemoglobin concentration) will be analysed using a likelihood-based longitudinal data analysis model.[27] Appropriate transformations may be applied to the variables before fitting the model if considered skewed (e.g. ferritin). Additional analyses using multiple imputation will be performed to handle missing data. Results will be compared with the main analysis to investigate the findings' robustness to the missing data assumptions. Safety, including adverse events, infections and clinic visits, will be presented for the mothers and neonates, respectively. The proportion of study participants with at least one safety outcome will be compared between groups using a log-binomial regression model. A Poisson model with robust standard errors will be fitted if there is a non-convergence of the log-binomial model for efficacy or safety outcomes. Exploratory subgroup analyses (e.g., by parity, site, iron deficiency) will be performed for maternal and neonatal outcomes, irrespective of their findings. The analyses models for all study outcomes will adjust for the randomisation stratification variable of the site as a main effect.

For peer review only

Data Management

Data collected from the subjects are recorded in digital Case Report Forms (CRFs) using tablets. Relevant hard copy patient hospital files are scanned for reference and stored digitally, securely. An independent Data and Safety Monitoring Board has been established to regularly review the trial's progress and blinded and unblinded results. The Research Support Centre at the College of Medicine performs independent monitoring of the study on behalf of the sponsor. No interim analysis is planned.

For peer review only

ETHICS AND DISSEMINATION

The College of Medicine Research Ethics Committee (COMREC), Blantyre, Malawi, and the Walter and Eliza Hall Institute of Medical Research Human Research Ethics Committee, Melbourne, Australia, approved this study. In addition, an evaluation was done by the Regional Ethics Committee West of Norway. They advised that the study was not subject to the Norwegian Health Research Act and that ethical review from this committee was not required. The trial is approved by the Malawian Pharmacy and Medicines Regulatory Authority (PMRA/CTRC/III/25052018100). Important protocol modifications such as changes to eligibility criteria or outcomes are reported to the ethics committees, regulatory authorities in Malawi, investigators, trial participants, trial registries. Witnessed informed written consent in English or Chichewa language is obtained from each participant before conducting any study-related procedure. The study is conducted under The International Conference on Harmonisation of Technical Requirements for Registration of Pharmaceuticals for Human Use (ICH) guidelines for "good clinical practice" (GCP) and the Declaration of Helsinki. The results will be presented to and shared with the local community that hosted and enabled the research, and also to

For peer review only - http://bmjopen.bmj.com/site/about/guidelines.xhtml

the international fora. We will publish in peer-reviewed scientific journals and report to relevant policymaking bodies such as the Malawi Ministry of Health.

DISCUSSION

Maternal anaemia prevalence is highest in sub-Saharan Africa and South Asia, and current approaches to control it are failing. This trial will provide high quality, African-based evidence for clinicians, policymakers and donors on a role for modern IV iron for antenatal anaemia in low-income settings. This is the largest RCT program assessing new IV iron formulations in pregnancy and is of major international significance in developing new global guidelines for anaemia in pregnancy. The results of this study will be disseminated to local and national medical authorities, policymakers, and be disseminated to the global research community, technical agencies, and international government bodies via peer-reviewed journals and at international scientific fora.

Author Contributions:

KSP, and S-RP were involved in conception and trial design. MNM wrote the first draft of the paper. MNM, GM, EM, SB, RH and RA were involved in drafting of the article. SB and RH provided statistical expertise. MNM, GM, EM, and KB were involved in study implementation and data acquisition. BR, JS, WS, SR, JC and LL were involved in critical revision of the article for important intellectual content. All the authors were involved in final approval of the article. Preparing study design, collection, management, analysis and interpretation of data; writing of the report; and the decision to submit the report for publication is the responsibility of the study sponsor. The study funder, the Bill and Melinda Gates Foundation, had no role in the decision to publish.

Funding statement: This study is funded by the Bill & Melinda Gates Foundation OPP1169939.

For peer review only - http://bmjopen.bmj.com/site/about/guidelines.xhtml

Acknowledgements:

We acknowledge all the study participants for their willingness to participate in this study. We appreciate the dedication of the research staff in Blantyre, Zomba and Melbourne. We are grateful for the support we continue to receive from the District Health Offices in Blantyre and Zomba districts of Malawi. We acknowledge the collaboration with the Zomba Central Hospital, especially the office of the Hospital Director. We thank Dr Alinune Kabaghe and Dr Josephine Banda, who independently packed the trial drugs and delivered them to research sites. We also acknowledge the Dr Kamiza Histopathology Laboratory for analysing the placental samples.

For peer review only

Competing interests statement.

None to declare.

Word Count: 4,962

For peer review only - http://bmjopen.bmj.com/site/about/guidelines.xhtml

References

1. World Health Organization. The global prevalence of anaemia in 2011. . In: World Health Organization, ed. Geneva, 2015.
2. Haider BA, Olofin I, Wang M, et al. Anaemia, prenatal iron use, and risk of adverse pregnancy outcomes: Systematic review and meta-analysis. *BMJ (Online)* 2013;347(7916) doi: 10.1136/bmj.f3443
3. Premru-Srsen T, Verdenik I, Ponikvar BM, et al. Infant mortality and causes of death by birth weight for gestational age in non-malformed singleton infants: a 2002-2012 population-based study. *J Perinat Med* 2018;46(5):547-53. doi: 10.1515/jpm-2017-0103 [published Online First: 2017/06/11]
4. World Health Organization, United Nations Children's Fund, United Nations University. Iron Deficiency Anaemia: Assessment, Prevention, and Control. A guide for programme managers. In: Organization. WH, ed. Geneva, 2001.
5. Daru J, Zamora J, Fernández-Félix BM, et al. Risk of maternal mortality in women with severe anaemia during pregnancy and post partum: a multilevel analysis. *Lancet Glob Health* 2018;6(5):e548-e54. doi: 10.1016/s2214-109x(18)30078-0 [published Online First: 2018/03/25]
6. Peña-Rosas JP, De-Regil LM, Garcia-Casal MN, et al. Daily oral iron supplementation during pregnancy. *Cochrane Database of Systematic Reviews* 2015;2015(7):1-527. doi: 10.1002/14651858.CD004736.pub5
7. Mwangi MN, Roth JM, Smit MR, et al. Effect of daily antenatal iron supplementation on plasmodium infection in kenyan women: A randomized clinical trial. *JAMA - Journal of the American Medical Association* 2015;314(10):1009-20. doi: 10.1001/jama.2015.9496
8. World Health Organization. Iron and folate supplementation. Integrated management of pregnancy and childbirth (IMPAC). . In: Organization WH, ed. Geneva, 2006.
9. World Health Organization. Essential Nutrition Actions: Improving maternal, newborn, infant and young child health and nutrition. . In: Organization. WH, ed. Geneva, 2013.
10. Low MSY, Speedy J, Styles CE, et al. Daily iron supplementation for improving anaemia, iron status and health in menstruating women. *Cochrane Database of Systematic Reviews* 2016;2016(4) doi: 10.1002/14651858.CD009747.pub2
11. Stoffel NU, Cercamondi CI, Brittenham G, et al. Iron absorption from oral iron supplements given on consecutive versus alternate days and as single morning doses versus twice-daily split dosing in iron-depleted women: two open-label, randomised controlled trials. *Lancet Haematol* 2017;4(11):e524-e33. doi: 10.1016/s2352-3026(17)30182-5 [published Online First: 2017/10/17]
12. National Statistical Office (NSO) [Malawi], ICF International. Malawi Demographic and Health Survey 2015-16: Key Indicators Report. In: NSO and ICF International, ed. Zomba, Malawi, and Rockville, Maryland, USA, 2016.
13. Bah A, Pasricha SR, Jallow MW, et al. Serum hepcidin concentrations decline during pregnancy and may identify iron deficiency: Analysis of a longitudinal pregnancy cohort in the Gambia. *Journal of Nutrition* 2017;147(6):1131-37. doi: 10.3945/jn.116.245373

For peer review only - http://bmjopen.bmj.com/site/about/guidelines.xhtml

14. Mkandawire P. Gestational Age at First Antenatal Care Visit in Malawi. *Matern Child Health J* 2015;19(11):2366-74. doi: 10.1007/s10995-015-1754-6 [published Online First: 2015/07/15]
15. Friedrisch JR, Cançado RD. Intravenous ferric carboxymaltose for the treatment of iron deficiency anemia. *Revista Brasileira de Hematologia e Hemoterapia* 2015;37(6):400-05. doi: 10.1016/j.bjhh.2015.08.012
16. Pasricha SR, Tye-Din J, Muckenthaler MU, et al. Iron deficiency. *Lancet* 2021;397(10270):233-48. doi: 10.1016/s0140-6736(20)32594-0 [published Online First: 2020/12/08]
17. Shand AW, Bell J, Henry A, et al. Rapid increase in intravenous iron therapy for women of reproductive age in Australia. *Med J Aust* 2020;213(2):85-86. doi: 10.5694/mja2.50618 [published Online First: 2020/05/27]
18. Qassim A, Grivell RM, Henry A, et al. Intravenous or oral iron for treating iron deficiency anaemia during pregnancy: systematic review and meta-analysis. *Med J Aust* 2019;211(8):367-73. doi: 10.5694/mja2.50308 [published Online First: 2019/08/24]
19. Achebe MM, Gafter-Gvili A. How I treat anemia in pregnancy: Iron, cobalamin, and folate. *Blood* 2017;129(8):940-49. doi: 10.1182/blood-2016-08-672246
20. Neogi SB, Devasenapathy N, Singh R, et al. Safety and effectiveness of intravenous iron sucrose versus standard oral iron therapy in pregnant women with moderate-to-severe anaemia in India: a multicentre, open-label, phase 3, randomised, controlled trial. *Lancet Glob Health* 2019;7(12):e1706-e16. doi: 10.1016/s2214-109x(19)30427-9 [published Online First: 2019/11/12]
21. Ortiz R, Toblli JE, Romero JD, et al. Efficacy and safety of oral iron(III) polymaltose complex versus ferrous sulfate in pregnant women with iron-deficiency anemia: a multicenter, randomized, controlled study. *J Matern Fetal Neonatal Med* 2011;24(11):1347-52. doi: 10.3109/14767058.2011.599080 [published Online First: 2011/08/24]
22. Vanobberghen F, Lweno O, Kuemmerle A, et al. Efficacy and safety of intravenous ferric carboxymaltose compared with oral iron for the treatment of iron deficiency anaemia in women after childbirth in Tanzania: a parallel-group, open-label, randomised controlled phase 3 trial. *Lancet Glob Health* 2021;9(2):e189-e98. doi: 10.1016/s2214-109x(20)30448-4 [published Online First: 2020/11/28]
23. World Bank. Prevalence of low birthweight babies (% of births) - Malawi Washington DC, USA2021 [21 August 2021]. Available from: https://data.worldbank.org/indicator/SH.STA.BRTW.ZS?locations=MW.
24. Boudová S, Divala T, Mawindo P, et al. The prevalence of malaria at first antenatal visit in Blantyre, Malawi declined following a universal bed net campaign. *Malar J* 2015;14:422. doi: 10.1186/s12936-015-0945-3 [published Online First: 2015/10/30]
25. Young MF, Oaks BM, Tandon S, et al. Maternal hemoglobin concentrations across pregnancy and maternal and child health: a systematic review and meta-analysis. *Ann N Y Acad Sci* 2019;1450(1):47-68. doi: 10.1111/nyas.14093 [published Online First: 2019/04/18]
26. Breymann C, Milman N, Mezzacasa A, et al. Ferric carboxymaltose vs. oral iron in the treatment of pregnant women with iron deficiency anemia: An international, open-label, randomized controlled trial (FER-ASAP). *Journal of Perinatal Medicine* 2017;45(4):443-53. doi: 10.1515/jpm-2016-0050

For peer review only

27. Liang K-Y, Zeger SL. Longitudinal data analysis of continuous and discrete responses for pre-post designs *Sankhyā: The Indian Journal of Statistics* 2000;Series B:134-48. doi: doi: https://www.jstor.org/stable/25053123

For peer review only

For peer review only - http://bmjopen.bmj.com/site/about/guidelines.xhtml

Figure heading, caption and legend

Figure 1

Figure caption and legend: *REVAMP Trial schema. V0 – visit 0; V1 – Visit 1; V2 – Visit 2; V4 – Visit 4; V7 – Visit 7; V8 – Visit 8; Abbreviations: iv – Intravenous; wks – weeks.*

For peer review only

For peer review only - http://bmjopen.bmj.com/site/about/guidelines.xhtml

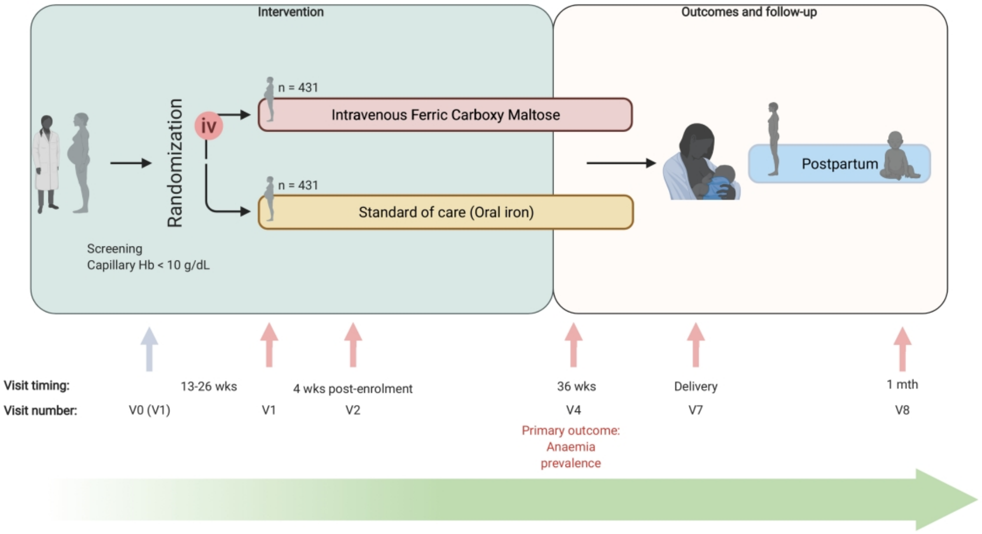

REVAMP Trial schema. V0 – visit 0; V1 – Visit 1; V2 – Visit 2; V4 – Visit 4; V7 – Visit 7; V8 – Visit 8; Abbreviations: iv – Intravenous; wks – weeks.

Reporting checklist for protocol of a clinical trial.

Based on the SPIRIT guidelines.

Instructions to authors

Complete this checklist by entering the page numbers from your manuscript where readers will find each of the items listed below.

Your article may not currently address all the items on the checklist. Please modify your text to include the missing information. If you are certain that an item does not apply, please write "n/a" and provide a short explanation.

Upload your completed checklist as an extra file when you submit to a journal.

In your methods section, say that you used the SPIRITreporting guidelines, and cite them as:

Chan A-W, Tetzlaff JM, Gøtzsche PC, Altman DG, Mann H, Berlin J, Dickersin K, Hróbjartsson A, Schulz KF, Parulekar WR, Krleža-Jerić K, Laupacis A, Moher D. SPIRIT 2013 Explanation and Elaboration: Guidance for protocols of clinical trials. BMJ. 2013;346:e7586

		Reporting Item	Page Number
**Administrative information**			1
Title	#1	Descriptive title identifying the study design, population, interventions, and, if applicable, trial acronym	1
Trial registration	#2a	Trial identifier and registry name. If not yet registered, name of intended registry	2 & 4
Trial registration: data set	#2b	All items from the World Health Organization Trial Registration Data Set	4
Protocol version	#3	Date and version identifier	4
Funding	#4	Sources and types of financial, material, and other support	26
Roles and responsibilities: contributorship	#5a	Names, affiliations, and roles of protocol contributors	1 & 2
Roles and responsibilities: sponsor contact information	#5b	Name and contact information for the trial sponsor	4
Roles and responsibilities:	#5c	Role of study sponsor and funders, if any, in study design; collection, management, analysis, and interpretation of data;	4

For peer review only

sponsor and funder		writing of the report; and the decision to submit the report for publication, including whether they will have ultimate authority over any of these activities	
Roles and responsibilities: committees	#5d	Composition, roles, and responsibilities of the coordinating centre, steering committee, endpoint adjudication committee, data management team, and other individuals or groups overseeing the trial, if applicable (see Item 21a for data monitoring committee)	19
**Introduction**			
Background and rationale	#6a	Description of research question and justification for undertaking the trial, including summary of relevant studies (published and unpublished) examining benefits and harms for each intervention	7
Background and rationale: choice of comparators	#6b	Explanation for choice of comparators	20
Objectives	#7	Specific objectives or hypotheses	9,10
Trial design	#8	Description of trial design including type of trial (eg, parallel group, crossover, factorial, single group), allocation ratio, and framework (eg, superiority, equivalence, non-inferiority, exploratory)	10
**Methods: Participants, interventions, and outcomes**			
Study setting	#9	Description of study settings (eg, community clinic, academic hospital) and list of countries where data will be collected. Reference to where list of study sites can be obtained	10,11
Eligibility criteria	#10	Inclusion and exclusion criteria for participants. If applicable, eligibility criteria for study centres and individuals who will perform the interventions (eg, surgeons, psychotherapists)	11,12
Interventions: description	#11a	Interventions for each group with sufficient detail to allow replication, including how and when they will be administered	12,13
Interventions: modifications	#11b	Criteria for discontinuing or modifying allocated interventions for a given trial participant (eg, drug dose change in response to harms, participant request, or improving / worsening disease)	13
Interventions: adherance	#11c	Strategies to improve adherence to intervention protocols, and any procedures for monitoring adherence (eg, drug tablet return; laboratory tests)	13

For peer review only

Interventions: concomitant care	#11d	Relevant concomitant care and interventions that are permitted or prohibited during the trial	13
Outcomes	#12	Primary, secondary, and other outcomes, including the specific measurement variable (eg, systolic blood pressure), analysis metric (eg, change from baseline, final value, time to event), method of aggregation (eg, median, proportion), and time point for each outcome. Explanation of the clinical relevance of chosen efficacy and harm outcomes is strongly recommended	21
Participant timeline	#13	Time schedule of enrolment, interventions (including any run-ins and washouts), assessments, and visits for participants. A schematic diagram is highly recommended (see Figure)	10,18, 19,20
Sample size	#14	Estimated number of participants needed to achieve study objectives and how it was determined, including clinical and statistical assumptions supporting any sample size calculations	22, 23
Recruitment	#15	Strategies for achieving adequate participant enrolment to reach target sample size	18
**Methods: Assignment of interventions (for controlled trials)**			
Allocation: sequence generation	#16a	Method of generating the allocation sequence (eg, computer-generated random numbers), and list of any factors for stratification. To reduce predictability of a random sequence, details of any planned restriction (eg, blocking) should be provided in a separate document that is unavailable to those who enrol participants or assign interventions	13
Allocation concealment mechanism	#16b	Mechanism of implementing the allocation sequence (eg, central telephone; sequentially numbered, opaque, sealed envelopes), describing any steps to conceal the sequence until interventions are assigned	13
Allocation: implementation	#16c	Who will generate the allocation sequence, who will enrol participants, and who will assign participants to interventions	13
Blinding (masking)	#17a	Who will be blinded after assignment to interventions (eg, trial participants, care providers, outcome assessors, data analysts), and how	13,14
Blinding (masking): emergency unblinding	#17b	If blinded, circumstances under which unblinding is permissible, and procedure for revealing a participant's allocated intervention during the trial	13,14

For peer review only

Methods: Data collection, management, and analysis

Data collection plan	#18a	Plans for assessment and collection of outcome, baseline, and other trial data, including any related processes to promote data quality (eg, duplicate measurements, training of assessors) and a description of study instruments (eg, questionnaires, laboratory tests) along with their reliability and validity, if known. Reference to where data collection forms can be found, if not in the protocol	20
Data collection plan: retention	#18b	Plans to promote participant retention and complete follow-up, including list of any outcome data to be collected for participants who discontinue or deviate from intervention protocols	20,21
Data management	#19	Plans for data entry, coding, security, and storage, including any related processes to promote data quality (eg, double data entry; range checks for data values). Reference to where details of data management procedures can be found, if not in the protocol	24
Statistics: outcomes	#20a	Statistical methods for analysing primary and secondary outcomes. Reference to where other details of the statistical analysis plan can be found, if not in the protocol	23
Statistics: additional analyses	#20b	Methods for any additional analyses (eg, subgroup and adjusted analyses)	n/a: statistical analysis plan to be published separately
Statistics: analysis population and missing data	#20c	Definition of analysis population relating to protocol non-adherence (eg, as randomised analysis), and any statistical methods to handle missing data (eg, multiple imputation)	23 (SAP to be published separately)
**Methods: Monitoring**			
Data monitoring: formal committee	#21a	Composition of data monitoring committee (DMC); summary of its role and reporting structure; statement of whether it is independent from the sponsor and competing interests; and reference to where further details about its charter can be found, if not in the protocol. Alternatively, an explanation of why a DMC is not needed	19
Data monitoring: interim analysis	#21b	Description of any interim analyses and stopping guidelines, including who will have access to these interim results and make the final decision to terminate the trial	23,24
Harms	#22	Plans for collecting, assessing, reporting, and managing solicited	21,22

For peer review only

		and spontaneously reported adverse events and other unintended effects of trial interventions or trial conduct	
Auditing	#23	Frequency and procedures for auditing trial conduct, if any, and whether the process will be independent from investigators and the sponsor	19
**Ethics and dissemination**			
Research ethics approval	#24	Plans for seeking research ethics committee / institutional review board (REC / IRB) approval	4
Protocol amendments	#25	Plans for communicating important protocol modifications (eg, changes to eligibility criteria, outcomes, analyses) to relevant parties (eg, investigators, REC / IRBs, trial participants, trial registries, journals, regulators)	4
Consent or assent	#26a	Who will obtain informed consent or assent from potential trial participants or authorised surrogates, and how (see Item 32)	See supplementary material 1
Consent or assent: ancillary studies	#26b	Additional consent provisions for collection and use of participant data and biological specimens in ancillary studies, if applicable	See supp. Material 1
Confidentiality	#27	How personal information about potential and enrolled participants will be collected, shared, and maintained in order to protect confidentiality before, during, and after the trial	23,24
Declaration of interests	#28	Financial and other competing interests for principal investigators for the overall trial and each study site	25
Data access	#29	Statement of who will have access to the final trial dataset, and disclosure of contractual agreements that limit such access for investigators	23,24
Ancillary and post trial care	#30	Provisions, if any, for ancillary and post-trial care, and for compensation to those who suffer harm from trial participation	21
Dissemination policy: trial results	#31a	Plans for investigators and sponsor to communicate trial results to participants, healthcare professionals, the public, and other relevant groups (eg, via publication, reporting in results databases, or other data sharing arrangements), including any publication restrictions	8,24
Dissemination policy: authorship	#31b	Authorship eligibility guidelines and any intended use of professional writers	24

For peer review only

Dissemination policy: reproducible research	#31c	Plans, if any, for granting public access to the full protocol, participant-level dataset, and statistical code	n/a; statistical analyses plan to be published separately
**Appendices**			
Informed consent materials	#32	Model consent form and other related documentation given to participants and authorised surrogates	Supplementary material 1
Biological specimens	#33	Plans for collection, laboratory evaluation, and storage of biological specimens for genetic or molecular analysis in the current trial and for future use in ancillary studies, if applicable	14,15, 16

None The SPIRIT Explanation and Elaboration paper is distributed under the terms of the Creative Commons Attribution License CC-BY-NC. This checklist can be completed online using https://www.goodreports.org/, a tool made by the EQUATOR Network in collaboration with Penelope.ai

For peer review only

BMJ Open

Protocol for a multicentre, parallel-group, open-label randomised controlled trial comparing ferric carboxymaltose with the standard of care in anaemic Malawian pregnant women – The REVAMP trial

Journal:	*BMJ Open*
Manuscript ID	bmjopen-2021-053288.R2
Article Type:	Protocol
Date Submitted by the Author:	14-Oct-2021
Complete List of Authors:	Mwangi, Martin; University of Malawi College of Medicine; Training and Research Unit of Excellence (TRUE), Nutrition and Infectious Diseases<br>Mzembe, Glory; University of Malawi College of Medicine, Training and Research Unit of Excellence (TRUE); University of Malawi College of Medicine, School of Public Health and Family Medicine, Department of Public Health<br>Moya, Ernest; University of Malawi College of Medicine, Training and Research Unit of Excellence (TRUE); University of Malawi College of Medicine, School of Public Health and Family Medicine, Department of Public Health<br>Braat, Sabine ; University of Melbourne School of Population and Global Health, Centre for Epidemiology and Biostatistics; The University of Melbourne Melbourne Medical School, Department of Infectious Diseases<br>Harding, Rebecca; The Walter and Eliza Hall Institute of Medical Research, Population Health and Immunity Division,<br>Robberstad, Bjarne; University of Bergen, Department of Global Public Health and Primary Care<br>Simpson, Julie; University of Melbourne School of Population and Global Health, Centre for Epidemiology and Biostatistics<br>Stones, William; University of Malawi, Obstetrics & Gynaecology<br>Rogerson, Stephen; University of Melbourne, Department of Medicine at the Peter Doherty Institute for Infection and Immunity; The University of Melbourne Melbourne Medical School, Department of Infectious Diseases<br>Biselele, Kabeya; Zomba Central Hospital<br>Chinkhumba, Jobiba; University of Malawi College of Medicine, Malaria Alert Centre<br>Larson, Leila; University of South Carolina Arnold School of Public Health, Department of Health Promotion, Education, and Behavior<br>Ataíde, Ricardo; The Walter and Eliza Hall Institute of Medical Research, Population Health and Immunity Division; The University of Melbourne, Department of Infectious Diseases<br>Phiri, Kamija; University of Malawi College of Medicine, Training and Research Unit of Excellence (TRUE); University of Malawi College of Medicine, School of Public Health and Family Medicine, Department of Public Health<br>Pasricha, Sant-Rayn; Walter and Eliza Hall Institute of Medical Research, Population Health and Immunity Division; The Royal Melbourne Hospital, Diagnostic Haematology and Clinical Haematology, The Peter MacCallum

	Cancer Centre
**Primary Subject Heading**:	Haematology (incl blood transfusion)
Secondary Subject Heading:	Global health, Nutrition and metabolism, Public health, Infectious diseases, Epidemiology
Keywords:	Anaemia < HAEMATOLOGY, Nutrition < TROPICAL MEDICINE, Epidemiology < TROPICAL MEDICINE, Clinical trials < THERAPEUTICS, PUBLIC HEALTH, Epidemiology < INFECTIOUS DISEASES

SCHOLARONE™
Manuscripts

I, the Submitting Author has the right to grant and does grant on behalf of all authors of the Work (as defined in the below author licence), an exclusive licence and/or a non-exclusive licence for contributions from authors who are: i) UK Crown employees; ii) where BMJ has agreed a CC-BY licence shall apply, and/or iii) in accordance with the terms applicable for US Federal Government officers or employees acting as part of their official duties; on a worldwide, perpetual, irrevocable, royalty-free basis to BMJ Publishing Group Ltd ("BMJ") its licensees and where the relevant Journal is co-owned by BMJ to the co-owners of the Journal, to publish the Work in this journal and any other BMJ products and to exploit all rights, as set out in our licence.

The Submitting Author accepts and understands that any supply made under these terms is made by BMJ to the Submitting Author unless you are acting as an employee on behalf of your employer or a postgraduate student of an affiliated institution which is paying any applicable article publishing charge ("APC") for Open Access articles. Where the Submitting Author wishes to make the Work available on an Open Access basis (and intends to pay the relevant APC), the terms of reuse of such Open Access shall be governed by a Creative Commons licence – details of these licences and which Creative Commons licence will apply to this Work are set out in our licence referred to above.

Other than as permitted in any relevant BMJ Author's Self Archiving Policies, I confirm this Work has not been accepted for publication elsewhere, is not being considered for publication elsewhere and does not duplicate material already published. I confirm all authors consent to publication of this Work and authorise the granting of this licence.

For peer review only

Protocol for a multicentre, parallel-group, open-label randomised controlled trial comparing ferric carboxymaltose with the standard of care in anaemic Malawian pregnant women – The REVAMP trial

Authors

Martin N Mwangi[1,2], Glory Mzembe[1,2], Ernest Moya[1,2], Sabine Braat[3,4,5], Rebecca Harding[3], Bjarne Robberstad[6], Julie Simpson[5], William Stones[2], Stephen Rogerson,[4,7] Kabeya Biselele[8], Jobiba Chinkhumba[9], Leila Larson[10], Ricardo Ataíde[3,4], Kamija S Phiri[1,2], Sant-Rayn Pasricha[3,11,12]

Institutions

1. Training and Research Unit of Excellence (TRUE), Mandala, No. 1 Kufa Road, Chichiri, BT3, Blantyre, Malawi.
2. School of Public Health and Family Medicine, Department of Public Health, Kamuzu University of Health Sciences (KUHeS), Chichiri, BT3, Blantyre, Malawi.
3. Population Health and Immunity Division, The Walter and Eliza Hall Institute of Medical Research, 1G Royal Parade, Parkville, Melbourne 3052, Australia.
4. Department of Infectious Diseases, Melbourne Medical School, The University of Melbourne, Melbourne, Australia.
5. Centre for Epidemiology and Biostatistics, Melbourne School of Population and Global Health, The University of Melbourne, Melbourne, Australia.
6. Department of Global Public Health and Primary Care, University of Bergen, Bergen, Norway.
7. Department of Medicine at the Peter Doherty Institute for Infection and Immunity, University of Melbourne, Melbourne, Australia.
8. Zomba Central Hospital, Department of Obstetrics and Gynecology, Zomba, Malawi.

For peer review only

9. Malaria Alert Centre, Kamuzu University of Health Sciences (KUHeS), Chichiri, BT3, Blantyre, Malawi.
10. Department of Health Promotion, Education, and Behaviour, Arnold School of Public Health, University of South Carolina, Columbia, South Carolina, USA.
11. Diagnostic Haematology, The Royal Melbourne Hospital; and Clinical Haematology, The Peter MacCallum Cancer Centre and The Royal Melbourne Hospital, Parkville VIC 3050 Australia.
12. Department of Medical Biology, Faculty of Medicine, Dentistry and Health Sciences, The University of Melbourne, Melbourne, Australia.

For peer review only

Protocol registry number: ACTRN12618001268235

Corresponding author:

Martin N. Mwangi, P.O. Box 30538, Chichiri, BT3, Blantyre, Malawi. Email: mmwangi@medcol.mw

Word count: 4,962 words. (Abstract) 299 words. *(excluding title page, abstract, references, figures and tables)*

For peer review only - http://bmjopen.bmj.com/site/about/guidelines.xhtml

ABSTRACT

Introduction: Anaemia in pregnancy remains a critical global health problem, affecting 46% of pregnant women in Africa and 49% in Asia. Oral iron therapy requires extended adherence to achieve correction of anaemia and replenishment of iron stores. Ferric carboxymaltose (FCM) is a recently established intravenous iron formulation associated with substantial advantages in safety, speed of delivery and total dose deliverable in a single infusion. We aim to determine whether FCM given once during the second trimester of pregnancy compared to standard oral iron distributed through routine antenatal services is effective and safe for treatment of moderate to severe maternal anaemia in sub-Saharan Africa.

Methods and analysis: REVAMP is a two-arm confirmatory individually-randomised controlled trial set in Blantyre and Zomba districts in Malawi. The trial will randomise 862 women in the second trimester of pregnancy with a capillary haemoglobin concentration below 10.0g/dL. The study comprises two arms: (a) intravenous ferric carboxymaltose (20mg/kg up to 1000mg) given once at randomisation, and (b) standard of care oral iron (65mg elemental iron twice daily) for 90 days (or the duration of pregnancy, whichever is shorter) provided according to local health care practices. Both arms receive sulfadoxine-pyrimethamine (SP) as Intermittent Preventive Treatment in pregnancy (IPTp). The primary outcome is the prevalence of anaemia (Hb<11.0g/dL) at 36 weeks' gestation. Secondary outcomes include birth weight, gestation duration, and safety outcomes, including clinical malaria, serious perinatal events and postpartum haematologic and health-related outcomes in the mother and child.

Ethics and dissemination: Ethical approval was granted by the Research Ethics Committee (COMREC P.02/18/2357) in Malawi and the Human Research Ethics Committee (WEHI: 18/02), Melbourne, Australia. The protocol is registered with the Australian and New Zealand Clinical Trials Registry

For peer review only - http://bmjopen.bmj.com/site/about/guidelines.xhtml

(ACTRN12618001268235). The results will be shared with the local community that enabled the research, and also to the international fora.

Keywords: Anaemia, Iron Deficiency, Intravenous Iron, ferric carboxymaltose (FCM), Pregnancy, Malawi, randomised controlled trial

Data Statement

All relevant data are within the paper and its supporting information files.

Protocol version

REVAMP Trial protocol version 5.0 dated 21 November 2020

Ethics approval

College of Medicine Research Ethics Committee (COMREC P.02/18/2357) in Malawi and the Walter and Eliza Hall Institute of Medical Research Human Research Ethics Committee (WEHI: 18/02), Melbourne, Australia.

Trial Sponsor

Director - Research Support Centre.

College of Medicine, Kamuzu University of Health Sciences (KUHeS), Private Bag 360, Chichiri, Blantyre 3, Malawi.

Email: rscdirector@medcol.mw

Roles and responsibilities of sponsor and funder:

The sponsor and funder had no role in study design. They will have no role nor ultimate authority in the collection, management, analysis, and interpretation of data; writing of the report; and the decision to submit the report for publication.

For peer review only

ARTICLE SUMMARY

Strengths and limitations of this study

Strengths

1. Eligibility for inclusion - moderate or severe anaemia - is assessed by capillary haemoglobin estimation, a method that could be deployed at the local health centre level.
2. The trial follows pregnant women and their babies through delivery and into the postpartum period enabling the assessment of antenatal and postnatal effects of the intervention.
3. This trial uses a modern intravenous iron formulation for the treatment of anaemia in pregnancy which enables a high dose iron infusion (up to 1000mg) to be infused in a single dose.
4. The trial will measure a broad range of hematologic, safety and clinical-efficacy outcomes.

Limitations

5. This trial is open-label, and participants will know the trial intervention to which they have been randomised.

For peer review only - http://bmjopen.bmj.com/site/about/guidelines.xhtml

INTRODUCTION

Anaemia during pregnancy remains a critical global health problem. Almost 40% of pregnant women worldwide are anaemic, including 46% of pregnant women in Africa and 49% in Asia.[1] Anaemia in pregnancy is very common and mostly results from iron deficiency and is associated with critical risks for both mother (e.g. life-threatening complications of postpartum haemorrhage) and child (especially prematurity and low birth weight,[2] which are associated with increased risk of neonatal and infant mortality,[3] and reduced iron stores in infancy with increased risk of subsequent anaemia and impaired development).[4] Severe anaemia in pregnancy is associated with a significantly increased risk of maternal mortality.[5] Worldwide, 15%-20% of births (>20 million annually, including 13% in sub-Saharan Africa) are low birthweight. At the same time, each year, over 1 million children die from preterm birth complications, making prevention and treatment of antenatal anaemia a crucial component of efforts to reduce low birth weight.

Systematic reviews confirm likely benefits from iron on maternal outcomes, including anaemia (70% reduction) and trends towards favourable infant outcomes, including increased birth weight and extended gestation duration.[6] A trial of oral iron compared with placebo in Kenyan pregnant women confirmed a benefit from iron on birth weight and gestation duration.[7] Global recommendations for managing anaemia in pregnancy in lower and middle income countries (LMICs) currently advise that women be treated with high-dose daily oral iron (120 mg of elemental iron) supplementation for three months.[8 9] However, oral iron is often poorly tolerated due to gastrointestinal adverse effects,[10] limiting adherence; lower doses with intermitted dosing have been used in non-pregnant women.[11] Delivery of iron during pregnancy requires high-quality performance of the primary health system. In Malawi, 10.6% of women were reported to take no iron during pregnancy, and 37.1% take less than 60 doses, with only 33.4% taking the recommended 90 or more doses.[12] Even when delivered, oral iron frequently fails to correct anaemia. For example, 61% of Gambian pregnant women who received iron at week 20 of gestation – when their Hb was lower than 10.0g/dL - still had a Hb<10.0g/dL at 30

For peer review only - http://bmjopen.bmj.com/site/about/guidelines.xhtml

weeks' gestation.[13] Finally, women may only present for their first antenatal visit late in the second trimester, curtailing the time available to optimise iron stores with oral iron to improve foetal development and risks associated with delivery; for example, in Malawi, fewer than 30% of pregnant women attend before the sixteenth week of gestation.[14]

Over the past decade, there have been dramatic improvements in parenteral (intravenous) iron therapeutics. Ferric carboxymaltose (FCM) is a widely used formulation that can be administered in a short period (15 min) and at large doses (up to 1000 mg) in a single infusion.[15 16] FCM has revolutionised the treatment of iron-deficiency anaemia in high-income country settings and is widely used in outpatient settings, emergency departments, and primary care.[17] In pregnancy, intravenous iron has been reported to be superior to oral iron in improving haemoglobin concentrations and may even increase birth weight.[18] Intravenous iron is increasingly recommended as a suitable first-line option for women with moderate or severe iron-deficiency anaemia beyond the first trimester of pregnancy.[19] Studies have evaluated the role of older forms of intravenous iron in pregnancy in low-income settings such as India,[20 21] whilst other studies have demonstrated the feasibility of using FCM in the post-partum period in sub-Saharan Africa[22] but modern formulations capable of delivering a rapid total-dose infusion have not yet been studied in women in pregnancy in low income countries.

It remains challenging to directly measure iron status in the field, as biomarkers generally require analysis using centralised laboratory infrastructure. In contrast, point-of-care measurement of capillary haemoglobin is a feasible field-friendly testing strategy that can be deployed with existing technology, of which HemoCue point-of-care devices are a good example. Screen-and-treat approaches for anaemia in pregnancy must rely on haemoglobin measurement until point-of-care assays for iron status become more widely available.

FCM presents a practical opportunity for rapid correction of moderate and severe antenatal anaemia

For peer review only

in a single dose, potentially improving maternal and neonatal clinical outcomes for pregnant women. It is, therefore, urgent to test this therapy in a low-income field setting where the burden of anaemia-related disease is heaviest. In Malawian pregnant women with moderate or severe anaemia during the second trimester of pregnancy, we aim to evaluate whether a single dose of intravenous ferric carboxymaltose is superior to oral iron provided via standard-of-care approaches.

METHODS AND ANALYSIS

Patient and Public Involvement

We held discussions with national policy stakeholders during the study planning stage, including the Ministry of Health. We discussed how the research might align with national research and health service priorities. Local community engagement was done via public meetings; the potential participants were first involved in the study's design during these meetings. We discussed the study with the traditional leaders, including traditional authorities (TAs), group village headmen (GVH), the village development committee (VDC), and ward councillors (political figures). The GVH were requested to cascade the information to the village chiefs who took the information to the community. Health workers were accessed via the District Health Offices in Blantyre and Zomba districts. Health workers from the participating health facilities, including the health surveillance assistants (HSAs) who directly work with the community, were informed about the proposed research and discussions on priorities beneficial to the community were discussed. The public, potential participants, and health workers identified malaria and anaemia as major public health issues in the community. Women outlined their experiences with iron supplements during their past pregnancies and identified the development of tolerable iron formulations as a research priority. Dissemination of findings at the national and community level will follow the schema used during study design and inception as outlined above.

For peer review only - http://bmjopen.bmj.com/site/about/guidelines.xhtml

Trial objectives

The primary objective of the trial is to determine whether, in Malawian women in the second trimester of pregnancy with moderate or severe anaemia, a single dose of FCM up to 1000mg is superior to standard-of-care (i.e. oral iron provided through local health services), in reducing maternal anaemia before delivery (at 36 weeks' gestation).

The secondary objectives are:

During pregnancy and up to one month postpartum, to determine the effects of FCM (compared with standard-of-care) on:

Effectiveness:

- Maternal haemoglobin concentration and iron status (measured through iron biomarkers), and
- Critical neonatal outcomes including birth weight (low birth weight), gestation duration (prematurity), small for gestational age and other perinatal outcomes.

Safety:

- Maternal and neonatal adverse events, including infection episodes, serious maternal complications, and hypophosphatemia.

Exploratory objectives up to one month postpartum for subsequent hypothesis generation are related to maternal cognition, depression, and fatigue, as well as costs of health care.

Study design

REVAMP is a multicentre, open-label, two-arm, parallel-group individually randomised controlled trial (RCT). An open-label (rather than blinded) design was selected as it was considered unfeasible to deliver the placebo intravenous infusions to Malawian pregnant women in this field-trial context (the

For peer review only

For peer review only - http://bmjopen.bmj.com/site/about/guidelines.xhtml

drug is dark-coloured). The trial recruited pregnant women in their second trimester and is following them until one month postpartum, after which we will report on the primary and secondary objectives. Extended follow-up of mothers and infants to 12-months postpartum is planned, as is a range of exploratory economic, biological and clinical outcomes. These analyses are beyond the scope of this protocol, as are the exploratory outcomes collected up to one month postpartum, and they will be reported separately. The trial design is summarised in **Figure 1**.

Study settings and participants

In Malawi, the average gestation age at the first antenatal clinic (ANC) visit is 22 weeks, with 61% presenting before 24 weeks. The 2015-16 DHS survey estimated a prevalence of anaemia (Hb < 11.0g/dL) in pregnant women in Malawi at 45.1%, including 22.4% of women with moderate or severe anaemia.[12] The prevalence of low birth weight in Malawi was around 14% in 2015.[23] Malawi is endemic for malaria and the prevalence of *Plasmodium spp.* parasitaemia at the first ANC visit in the study site exceeds 15%.[24] About 87% of women consume at least one dose of intermittent preventive treatment during pregnancy (IPTp), but this falls to 30% for women taking three or more doses.[12]

The REVAMP study has two major research sites namely Limbe Health Centre and Zomba Central Hospital located in Blantyre and Zomba districts respectively, in Southern Malawi. The two major sites act as the study coordination centres in the respective districts. The Blantyre site serves as the base for five urban health centres/antenatal clinics, whilst the Zomba site serves as the base for nine (four urban and five rural) health centres/antenatal clinics. Women are screened at the antenatal clinics and referred to the coordinating research site for enrolment, treatment and follow-up visits. Deliveries occur in government-operated birth suites attached to the study site with referral to a district hospital where required for obstetric indications.

For peer review only - http://bmjopen.bmj.com/site/about/guidelines.xhtml

Eligibility criteria

Women are only randomised if they fulfil all inclusion criteria and none of the exclusion criteria.

Inclusion criteria:

1. Confirmed singleton pregnancy at 13-26 weeks' gestation
2. Moderate to severe anaemia (capillary haemoglobin <10.0g/dL measured by HemoCue 301+) not clinically deemed to require an immediate blood transfusion. We excluded women with mild anaemia as thresholds to distinguish mild anaemia from health are indistinct, and because moderate and severe anaemia have an increased link to adverse maternal and child health outcomes.[25]
3. Negative malaria parasitaemia at screening and no positive malaria rapid diagnostic test within the previous seven days
4. Resident in the study catchment area of Blantyre and Zomba district
5. Plan to deliver at a health facility
6. Written informed consent, including assent if <18 years old (consent of a parent or guardian plus the participant's assent).

Exclusion criteria:

1. Hypersensitivity to any of the study drugs
2. Clinical symptoms of malaria or positive malaria rapid diagnostic test within the previous seven days, or symptoms of bacterial infection, at screening
3. Any condition requiring hospitalisation or serious concomitant illness
4. Chronic illness that may adversely affect foetal growth and viability
5. Severe anaemia clinically judged as requiring a blood transfusion (usually Hb <5.0g/dL)
6. Pre-eclampsia at screening (new onset of hypertension, proteinuria and swelling in the legs, feet, and hands).

For peer review only

Trial Interventions

Participants are randomly assigned to receive one of the following interventions:

1. **Intravenous iron**: ferric carboxymaltose 20mg/kg up to 1000mg, diluted in normal saline and given over 15 minutes, once after randomisation. The study clinician administers the drug, and women remain under observation for 45 minutes after drug administration. Women receiving FCM are treated in rooms equipped with a trolley containing adrenaline, hydrocortisone, intravenous fluids and antihistamines, airway equipment and an oxygen concentrator. Staff are trained in the management of allergic reactions. Ferric carboxymaltose, manufactured by Vifor Pharma, was purchased at full-price from Aspen Pharma in Australia and shipped to Malawi.
2. **Standard-of-care - Oral iron treatment course**: oral iron- 200mg ferrous sulphate (approx. 65mg elemental iron) twice daily for 90 days or the remaining duration of pregnancy, whichever is shorter. Oral iron is provided by health workers using the same education messaging and similar packaging as provided by the routine health system to reflect real-life health service conditions.

Following national guidelines, both groups receive IPTp with 1500 mg Sulfadoxine and 75 mg Pyrimethamine (SP), as three tablets of SP of fixed dose (500mg/25mg) at least three times during pregnancy, at least four weeks apart. The IPTp-SP course at four weeks' post-randomisation is directly observed by study staff. HIV positive women in Malawi are not offered SP because they are already on Cotrimoxazole prophylaxis. The baseline dose is also directly administered by study staff if the participant did not receive it through the health service. All participants also receive an insecticide-treated bed net.

Randomisation, allocation concealment, and blinding

Participants are randomly allocated to one of the two treatments arms with 1:1 allocation via a computer-generated randomisation schedule of randomly permuted blocks stratified by research site

For peer review only

(Blantyre, Zomba) to achieve a balance between the arms within each site. The randomisation list was generated by an independent statistician at the University of Melbourne (Australia).

Individual participant codes were pre-packed in sealed envelopes by an independent researcher not associated with the study and held securely at the research sites. Eligible participants who meet all inclusion/exclusion criteria are sequentially allocated participant identification numbers within the research site, and their allocation to study group is revealed after opening the corresponding sealed envelope. Although the trial is open-label, laboratory scientists measuring haemoglobin concentration, midwives collecting birth outcome data, and investigators and researchers in Australia (including data managers and statisticians in Melbourne) are blinded to the treatment allocation during the conduct of the trial until the database is locked and ready for unblinding.

Recruitment and Visits

Table 1 shows the schedule of activities per visit for the mother, and **Table 2** shows the schedule of activities per visit for the neonate.

For peer review only

Table 1: Planned activities per visit for mothers

**Protocol activity**	**Visit 0**	**Visit 1**	**Visit 2**	**Visit 3**[j]	**Visit 4**	**Visit 5 – 6**[j]	**Visit 7**	**Visit 8**
	Day -7 to 0	Day 0	Day 28 ±2 days	34 wks. gestation ±2 days	36 wks. gestation ±2 days	38-40 wks. gestation ± 2 days	Delivery + 1 day	Day 28 postpartum ±2 days

For peer review only

Location of visit	*Antenatal Clinic*	*Research Site[a]*	*Research Site[a]*	*Home[b]*	*Research Site[a]*	*Home[b]*	*Research Site[a]*	*Research Site[a]*
Informed consent process for pre-screening	X							
Pre-screening	X							
Detailed Informed consent process	X	X						
Screening		X						
Medical & obstetric history		X						
Demographics		X						
Maternal physical examination[d]		X[h]	X[i]		X[i]		X[h]	X[i]
Ultrasound scan **(Fetal Biometry)[d]**		X	X		X			
Randomise participant		X						
Administer treatment		X						
Intermittent preventive treatment[c]		X	X		X			
Laboratory procedures								
Capillary haemoglobin[d]	X	X	X	X	X	X	X	X
Full blood count[d]		X	X		X		X	X
Malaria diagnostics[d, e]	X	X	X		X		X	X
Serum for iron markers tests[f]		X	X		X		X	X

For peer review only

Serum for inflammatory markers tests[g]		X	X		X		X	X
Phosphate		X	X		X		X	X
Adverse events[d]		X	X		X		X	X
Morbidities								X
Missed visits[d]			X	X	X	X	X	X
End of study			X	X	X	X	X	X

[a] Visit conducted at the research site/health facility; [b] Visit conducted at the participant's home; [c] Assessing if intermittent preventive treatments sulphadoxine pyrimethamine and albendazole were given; [d] Protocol activities are also collected at any unscheduled visits; [e] Malaria diagnostics include malaria rapid diagnostic test, malaria microscopy, [f] Serum for iron markers tests include serum ferritin; [g] Serum for inflammatory markers tests include C-reactive protein and alpha-1 glycoprotein; [h] Complete maternal physical examination includes general appearance, throat, neck, thyroid, musculoskeletal, skin, lymph nodes, extremities, pulses, pulmonary, cardiac, abdominal, and neurological examination; [i] Limited maternal physical examination includes general appearance, brief pulmonary, cardiac, abdominal and neurological examination. [j] Since the opening of the trial, visits 3, 5 and 6 have been suspended since April 2020 due to the risk of exposing communities to COVID-19 during home visits.

For peer review only

For peer review only - http://bmjopen.bmj.com/site/about/guidelines.xhtml

Table 2: Details of planned activities per visit for neonates

**Protocol activity**	**Visit 7**	**Visit 8**
	Delivery +1 day	Day 28 Postpartum ±2 days
Location of visit	*Research Site[a]*	*Research Site[a]*
Pregnancy outcome	X	
Physical examination and anthropometry [b, c]	X[f]	X[g]
Laboratory procedures		
Capillary haemoglobin	X[h]	
Full blood count[b]	X[h]	X
Malaria diagnostics[d]	X[h]	X
Serum for iron markers tests[e]	X[h]	X
Vaccination and Vit A supplementation status		X
Adverse events[b]	X	X
Morbidities		X
Missed visits[b]		X
End of study	X	X

[a] Visit done at the research site/ health facility; [b] Protocol activities are also collected at any unscheduled visits; [c] Anthropometry includes assess the infant's weight, length, and head circumference; [d] Malaria diagnostics include malaria rapid diagnostic test, malaria microscopy, malaria filter paper for polymerase chain reaction and histology at delivery; [e] Serum for iron markers tests include serum ferritin; [f] The complete physical examination includes general appearance, throat, neck, thyroid, musculoskeletal, skin, lymph nodes, extremities, pulses, pulmonary, cardiac, abdominal, and neurological examination [g] The limited examination includes general appearance, brief pulmonary, cardiac, abdominal and neurological examination; [h] These assessments are collected via cord blood.

For peer review only - http://bmjopen.bmj.com/site/about/guidelines.xhtml

Screening and enrolment of participants

Study recruitment opened on November 12, 2018; the final participant was recruited on March 02, 2021. Study activities are detailed in Tables 1 and 2.

Pre-screening and screening, visit 0 (day -7 to 0)

Women attending their first antenatal visit at ANCs are given general information (aims and procedures) about the study by a trained research nurse and invited to consent to pre-screening procedures. As the ANC nurse cares for attendees, the research nurse pre-screens potential participants by noting apparent gestation age based on clinical examination and last menstrual period and then tests for anaemia with capillary Hb (HemoCue 301+, Angelholm Sweden) and *Plasmodium* parasitaemia by rapid diagnostic test (SD Bioline Malaria AG P.F/PAN, Standard Diagnostics, Inc.). Potentially eligible participants receive further information about the study. Willing participants are referred to the research site for additional evaluation, full consenting procedure and recruitment.

Enrolment, visit 1 (day 0)

As presented in Table 1, at the health facility, potentially eligible participants undergo ultrasound scanning (within two days from the initial pre-screening visit) to confirm singleton pregnancy and determine gestation age (i.e. crown-rump length for less than or equal to 16 weeks, femur length and head and abdominal circumference for more than 16 weeks). Written informed consent is then obtained for participation in the trial. For consenting participants, a physical examination is conducted, and baseline data are collected, comprising demographic information and medical and obstetric history. Following the collection of baseline clinical data, samples of both capillary and venous blood are collected and then participants are randomised. For women randomised to the ferric carboxymaltose arm of the trial, an intravenous cannula is inserted under aseptic precautions, and a trained nurse administers the study drug according to standard procedures and under the supervision of the study clinician. Women randomised to the standard-of-care arm of the trial are provided with

For peer review only - http://bmjopen.bmj.com/site/about/guidelines.xhtml

a full course of iron tablets, together with information delivered according to a standardised script reflecting ANC practice.

Subsequent study visits

Visit 2 (28 (±2) days after enrolment into the study): Participants receive a physical examination and an ultrasound scan before being asked for a venous blood sample (Table 1). A capillary sample is collected for assessment of *Plasmodium* parasitaemia. The participants are asked to continue their regular ongoing ANC visits through their local health centre.

Visit 3; visits 5-6 (34 weeks' gestation ±2 days; 38-40 weeks' gestation ±2 days): Participants are visited in their home at 34 weeks' gestation, and every two weeks from 38 weeks' gestation until delivery. A research nurse measures capillary Hb. Where the participant belongs to the standard-of-care trial-arm, the research nurse attempts to assess adherence. Notably, since the opening of the trial, fieldwork has been affected by the COVID-19 pandemic. Because of the risks of study workers introducing COVID-19 into remote villages, home visits were removed from the trial protocol after April 2020.

Visit 4 (36 weeks' gestation/pre-delivery ±2 days): Procedures are similar to those for Visit 2 (28-days post-treatment). This visit includes the assessment of venous haemoglobin for the primary outcome.

Visit 7 (Delivery +1 day): The study provides 24-hour cover of the study research sites' delivery suites. All participants are asked to return to the research site for delivery (unless a high-risk pregnancy requires delivery at the tertiary referral hospital). Standardised delayed cord clamping procedures are instituted. Participants delivering at home or at other health facilities are encouraged to attend the research site for assessment within 24 hours. Venous blood samples are collected during labour. Apgar scores are recorded immediately after delivery, and the newborn undergoes a full physical examination, including measurement of birth weight, length, head circumference, and assessment for congenital malformations. The type of birth and occurrence of perinatal complications (including

For peer review only - http://bmjopen.bmj.com/site/about/guidelines.xhtml

haemorrhage or need for blood transfusion) are recorded. Placental blood is drawn, and a sample of placental tissue is collected and stored in buffered formalin for subsequent histological evaluation.

Visit 8 (28 days postpartum ±2 days): Participants return to the research site together with their infants for a detailed medical examination of both mother and infant and collection of blood samples.

Unscheduled visit: Participants are asked to attend the research site when symptomatically unwell. They are managed according to national standard treatment guidelines by a trained health care provider. Blood samples for malaria RDT (and microscopy if RDT positive) and blood culture are taken if clinically indicated. The clinical diagnosis of the unscheduled visit is recorded in the participant's record. Where a participant attends another health facility or antenatal clinic, the research team extracts the missed unscheduled visit notes from the participants health book commonly known as a health "passport" in Malawi. Usually, clinical investigations and medications are indicated in the health book and can be extracted by the research team during the next scheduled visit.

Laboratory Procedures

Venous blood is measured for haemoglobin concentration using an automated analyser (Sysmex, XP 300 series, Sysmex Corporation, Kobe, Japan), for which daily two level controls are run and recorded. Serum is separated by centrifugation and stored at -80 degrees Celsius. Samples will be batched and assayed for ferritin, C-reactive protein, and phosphate in Meander Medical Centre laboratory, accreditation number M040, EN ISO 15189:2012 (Amersfoort, The Netherlands).

Data Monitoring Committee (DMC)

An independent DMC has been set up to review on a regular basis, safety and efficacy data of the ongoing trial. The DMC is comprised of international experts in clinical trials, obstetrics, epidemiology and statistics. In the advent of clear evidence of effectiveness or harm, the DMC will recommend to the sponsor and investigators whether to continue, modify, or terminate the trial on ethical grounds.

For peer review only - http://bmjopen.bmj.com/site/about/guidelines.xhtml

For peer review only

Outcomes

The primary outcome is the prevalence of maternal anaemia before delivery, defined as a venous haemoglobin concentration less than 11.0g/dL at 36 weeks' gestation. This outcome evaluates the performance of the study intervention in helping women reach labour with optimal tissue oxygenation and resilience.

Secondary outcomes assessing effectiveness in the mother are anaemia at four weeks' post-randomisation and one month postpartum, moderate/ severe anaemia, haemoglobin concentration, iron deficiency (ferritin<15mg/L adjusted for C-reactive protein), iron deficiency anaemia, and serum ferritin concentration all at four weeks post-randomisation, 36 weeks' gestation, and one month postpartum.

Effectiveness in the neonate is assessed using the outcome of mean birthweight (measured in grams within 24 hours of birth) and the secondary outcomes low birth weight (birth weight less than 2500g), gestation duration (weeks), birth length, small for gestation age, fetal loss (pregnancy loss before 28 completed weeks' gestation), stillbirth (birth of a child showing no signs of life after 28 weeks' gestation), preterm births (neonate born before 37 completed weeks of gestation), haemoglobin concentration at 1-month postpartum and infant growth (weight for age, length for age, weight for length) at one month postpartum.

Outcomes assessing safety in the mother are (serious) adverse events related to administration of FCM (events occurring during the enrolment visit), (serious) adverse events (within 14 days of randomisation, antenatal and postpartum reported separately), all-cause sick visits to the clinic (antenatal and postpartum reported separately), clinical malaria-specific visits to the clinic (antenatal and postpartum reported separately), *P. falciparum* parasitaemia (at four weeks' post-randomisation and 36 weeks' gestation), hypophosphatemia (biochemical) (at four weeks' post-randomisation and 36 weeks' gestation), inflammation (elevated C-reactive protein) (at four weeks'

For peer review only

post-randomisation and 36 weeks' gestation), and severe medical events including haemorrhage, receipt of blood transfusion, ICU admission, or mortality.

Clinical infections will be reported during unplanned visits. Clinical malaria will be defined clinically, in women who present with fever and a positive malaria test. Diarrhoea will be defined in women with more than three loose stools per day. Other clinical diagnoses will be made according to local health manuals.

Safety in the infant is assessed using (serious) adverse events, all-cause visits to the clinic, infection-related visits to the clinic, diarrhoea-related visits to the clinic, and clinical malaria-specific visits to the clinic.

In addition to the primary and secondary clinical outcomes listed above, which will support the reporting of the confirmatory RCT, data for many exploratory outcomes are being collected between enrolment and 12-months postpartum. These include assessing maternal cognition, mother-infant bonding, maternal depression and fatigue, infant neurodevelopment, infant diet and health economic data, including direct and indirect costs of health care. Also, women have given their consent for the collection of samples for future translational studies, including evaluation of the vaginal and faecal microbiome, maternal immune profiles, and effects of interventions directed towards *P. falciparum* biology. Details of primary, secondary and exploratory outcomes included in the trial registration are shown in Supplementary Table 1.

For peer review only

Detection and reporting of Adverse Events and Serious Adverse Events

Non-serious Adverse Events (AEs) and serious adverse events (SAEs) are collected from the time consent is given until the participant completes the study (the final visit or withdrawal). These are detected either through spontaneous reports by the participant, unplanned visits to the research site or any of the participating health centres, observation by the study staff, and through standard

For peer review only - http://bmjopen.bmj.com/site/about/guidelines.xhtml

questioning at each visit. All SAEs are reported to the sponsor and the appropriate ethics committee, whether or not considered causally related to the study drugs.

Sample size

The sample size calculation is based on the primary maternal outcome of the proportion of women with pre-delivery anaemia (defined as venous Hb<11.0g/dL at 36 weeks' gestation). A systematic review and meta-analysis identified a 50% reduction in anaemia prevalence in women receiving oral iron.[2] In a cohort of pregnant women in the Gambia, we observed that 60% of pregnant women with Hb<10.0g/dL at 20 weeks' gestation who then receive iron interventions continue to have Hb<10.0g/dL by 30 weeks.[13] The pivotal Fer-ASAP trial (which compared FCM to oral iron in women with iron deficiency anaemia in high-income settings) demonstrated a 14% reduction in absolute anaemia prevalence compared with oral iron.[26] We hypothesise that routine iron supplementation will reduce anaemia prevalence from 100% to 60% (as seen in the Gambia,[13] and similar to data from Haider and colleagues[2]). We hypothesise that ferric carboxymaltose will result in a 10% absolute improvement in anaemia cure (to a prevalence of 50%). A total sample size of 862 pregnant women or 431 per group will provide 80% power to detect this difference, allowing for a 10% loss to follow-up and assuming a two-sided alpha of 5%. The sample size also has at least 80% power to detect an absolute difference between standard-of-care oral iron and ferric carboxymaltose of 100g in the neonatal outcome of birth weight (assuming a standard deviation of 450g and a two-sided alpha of 5%), similar to the effect size seen in a trial of Kenyan women receiving oral iron when compared to placebo.[7]

STATISTICAL ANALYSIS PLAN

A detailed statistical analysis plan will be finalised before unblinding of the database. Analyses will be performed where participants are classified according to their randomised intervention arm (i.e.

For peer review only - http://bmjopen.bmj.com/site/about/guidelines.xhtml

intention to treat principle). An available case analysis will be performed for repeated time point outcomes (e.g. anaemia) and a complete-case analysis for single time point outcomes (e.g. birth weight). Anaemia will be analysed using a log-binomial regression model, including study participants as a random intercept to account for the multiple time points. The model will include the standard-of-care (oral iron) group as the reference group. The primary maternal hypothesis will be evaluated by obtaining the estimate of the prevalence ratio of IV iron versus standard-of-care (oral iron), 95% confidence interval extracted at 36 weeks' gestation, and p-value. Birthweight will be analysed by fitting a linear regression model. The primary neonatal hypothesis will be evaluated by estimating the absolute difference in mean birth weight between IV iron and standard-of-care (oral iron) along with a corresponding 95% confidence interval and p-value. Secondary repeated time point binary outcomes will be analysed similarly to anaemia, and secondary single time point continuous outcomes will be analysed similarly to birthweight. Secondary, single time point binary outcomes (e.g., sub-optimal pregnancy outcomes) will be analysed using a log-binomial regression model and secondary, multiple time point continuous outcomes (e.g., maternal haemoglobin concentration) will be analysed using a likelihood-based longitudinal data analysis model.[27] Appropriate transformations may be applied to the variables before fitting the model if considered skewed (e.g. ferritin). Additional analyses using multiple imputation will be performed to handle missing data. Results will be compared with the main analysis to investigate the findings' robustness to the missing data assumptions. Safety, including adverse events, infections and clinic visits, will be presented for the mothers and neonates, respectively. The proportion of study participants with at least one safety outcome will be compared between groups using a log-binomial regression model. A Poisson model with robust standard errors will be fitted if there is a non-convergence of the log-binomial model for efficacy or safety outcomes. Exploratory subgroup analyses (e.g., by parity, site, iron deficiency) will be performed for maternal and neonatal outcomes, irrespective of their findings. The analyses models for all study outcomes will adjust for the randomisation stratification variable of the site as a main effect.

For peer review only

Data Management

Data collected from the subjects are recorded in digital Case Report Forms (CRFs) using tablets. Relevant hard copy patient hospital files are scanned for reference and stored digitally, securely. An independent Data and Safety Monitoring Board has been established to regularly review the trial's progress and blinded and unblinded results. The Research Support Centre at the College of Medicine performs independent monitoring of the study on behalf of the sponsor. No interim analysis is planned.

ETHICS AND DISSEMINATION

The College of Medicine Research Ethics Committee (COMREC), Blantyre, Malawi, and the Walter and Eliza Hall Institute of Medical Research Human Research Ethics Committee, Melbourne, Australia, approved this study. In addition, an evaluation was done by the Regional Ethics Committee West of Norway. They advised that the study was not subject to the Norwegian Health Research Act and that ethical review from this committee was not required. The trial is approved by the Malawian Pharmacy and Medicines Regulatory Authority (PMRA/CTRC/III/25052018100). Important protocol modifications such as changes to eligibility criteria or outcomes are reported to the ethics committees, regulatory authorities in Malawi, investigators, trial participants, trial registries. Witnessed informed written consent in English or Chichewa language is obtained from each participant before conducting any study-related procedure. The study is conducted under The International Conference on Harmonisation of Technical Requirements for Registration of Pharmaceuticals for Human Use (ICH) guidelines for "good clinical practice" (GCP) and the Declaration of Helsinki. The results will be presented to and shared with the local community that hosted and enabled the research, and also to

For peer review only

For peer review only - http://bmjopen.bmj.com/site/about/guidelines.xhtml

the international fora. We will publish in peer-reviewed scientific journals and report to relevant policymaking bodies such as the Malawi Ministry of Health.

DISCUSSION

Maternal anaemia prevalence is highest in sub-Saharan Africa and South Asia, and current approaches to control it are failing. This trial will provide high quality, African-based evidence for clinicians, policymakers and donors on a role for modern IV iron for antenatal anaemia in low-income settings. This is the largest RCT program assessing new IV iron formulations in pregnancy and is of major international significance in developing new global guidelines for anaemia in pregnancy. The results of this study will be disseminated to local and national medical authorities, policymakers, and be disseminated to the global research community, technical agencies, and international government bodies via peer-reviewed journals and at international scientific fora.

Author Contributions:

KSP, and S-RP were involved in conception and trial design. MNM wrote the first draft of the paper. MNM, GM, EM, SB, RH and RA were involved in drafting of the article. SB and RH provided statistical expertise. MNM, GM, EM, and KB were involved in study implementation and data acquisition. BR, JS, WS, SR, JC and LL were involved in critical revision of the article for important intellectual content. All the authors were involved in final approval of the article. Preparing study design, collection, management, analysis and interpretation of data; writing of the report; and the decision to submit the report for publication is the responsibility of the study sponsor. The study funder, the Bill and Melinda Gates Foundation, had no role in the decision to publish.

Funding statement: This study is funded by the Bill & Melinda Gates Foundation OPP1169939.

For peer review only - http://bmjopen.bmj.com/site/about/guidelines.xhtml

Acknowledgements:

We acknowledge all the study participants for their willingness to participate in this study. We appreciate the dedication of the research staff in Blantyre, Zomba and Melbourne. We are grateful for the support we continue to receive from the District Health Offices in Blantyre and Zomba districts of Malawi. We acknowledge the collaboration with the Zomba Central Hospital, especially the office of the Hospital Director. We thank Dr Alinune Kabaghe and Dr Josephine Banda, who independently packed the trial drugs and delivered them to research sites. We also acknowledge the Dr Kamiza Histopathology Laboratory for analysing the placental samples.

Competing interests statement.

None to declare.

Word Count: 4,962

For peer review only

References

1. World Health Organization. The global prevalence of anaemia in 2011. . In: World Health Organization, ed. Geneva, 2015.
2. Haider BA, Olofin I, Wang M, et al. Anaemia, prenatal iron use, and risk of adverse pregnancy outcomes: Systematic review and meta-analysis. *BMJ (Online)* 2013;347(7916) doi: 10.1136/bmj.f3443
3. Premru-Srsen T, Verdenik I, Ponikvar BM, et al. Infant mortality and causes of death by birth weight for gestational age in non-malformed singleton infants: a 2002-2012 population-based study. *J Perinat Med* 2018;46(5):547-53. doi: 10.1515/jpm-2017-0103 [published Online First: 2017/06/11]
4. World Health Organization, United Nations Children's Fund, United Nations University. Iron Deficiency Anaemia: Assessment, Prevention, and Control. A guide for programme managers. In: Organization. WH, ed. Geneva, 2001.
5. Daru J, Zamora J, Fernández-Félix BM, et al. Risk of maternal mortality in women with severe anaemia during pregnancy and post partum: a multilevel analysis. *Lancet Glob Health* 2018;6(5):e548-e54. doi: 10.1016/s2214-109x(18)30078-0 [published Online First: 2018/03/25]
6. Peña-Rosas JP, De-Regil LM, Garcia-Casal MN, et al. Daily oral iron supplementation during pregnancy. *Cochrane Database of Systematic Reviews* 2015;2015(7):1-527. doi: 10.1002/14651858.CD004736.pub5
7. Mwangi MN, Roth JM, Smit MR, et al. Effect of daily antenatal iron supplementation on plasmodium infection in kenyan women: A randomized clinical trial. *JAMA - Journal of the American Medical Association* 2015;314(10):1009-20. doi: 10.1001/jama.2015.9496
8. World Health Organization. Iron and folate supplementation. Integrated management of pregnancy and childbirth (IMPAC). . In: Organization WH, ed. Geneva, 2006.
9. World Health Organization. Essential Nutrition Actions: Improving maternal, newborn, infant and young child health and nutrition. . In: Organization. WH, ed. Geneva, 2013.
10. Low MSY, Speedy J, Styles CE, et al. Daily iron supplementation for improving anaemia, iron status and health in menstruating women. *Cochrane Database of Systematic Reviews* 2016;2016(4) doi: 10.1002/14651858.CD009747.pub2
11. Stoffel NU, Cercamondi CI, Brittenham G, et al. Iron absorption from oral iron supplements given on consecutive versus alternate days and as single morning doses versus twice-daily split dosing in iron-depleted women: two open-label, randomised controlled trials. *Lancet Haematol* 2017;4(11):e524-e33. doi: 10.1016/s2352-3026(17)30182-5 [published Online First: 2017/10/17]
12. National Statistical Office (NSO) [Malawi], ICF International. Malawi Demographic and Health Survey 2015-16: Key Indicators Report. In: NSO and ICF International, ed. Zomba, Malawi, and Rockville, Maryland, USA, 2016.
13. Bah A, Pasricha SR, Jallow MW, et al. Serum hepcidin concentrations decline during pregnancy and may identify iron deficiency: Analysis of a longitudinal pregnancy cohort in the Gambia. *Journal of Nutrition* 2017;147(6):1131-37. doi: 10.3945/jn.116.245373

For peer review only - http://bmjopen.bmj.com/site/about/guidelines.xhtml

14. Mkandawire P. Gestational Age at First Antenatal Care Visit in Malawi. *Matern Child Health J* 2015;19(11):2366-74. doi: 10.1007/s10995-015-1754-6 [published Online First: 2015/07/15]
15. Friedrisch JR, Cançado RD. Intravenous ferric carboxymaltose for the treatment of iron deficiency anemia. *Revista Brasileira de Hematologia e Hemoterapia* 2015;37(6):400-05. doi: 10.1016/j.bjhh.2015.08.012
16. Pasricha SR, Tye-Din J, Muckenthaler MU, et al. Iron deficiency. *Lancet* 2021;397(10270):233-48. doi: 10.1016/s0140-6736(20)32594-0 [published Online First: 2020/12/08]
17. Shand AW, Bell J, Henry A, et al. Rapid increase in intravenous iron therapy for women of reproductive age in Australia. *Med J Aust* 2020;213(2):85-86. doi: 10.5694/mja2.50618 [published Online First: 2020/05/27]
18. Qassim A, Grivell RM, Henry A, et al. Intravenous or oral iron for treating iron deficiency anaemia during pregnancy: systematic review and meta-analysis. *Med J Aust* 2019;211(8):367-73. doi: 10.5694/mja2.50308 [published Online First: 2019/08/24]
19. Achebe MM, Gafter-Gvili A. How I treat anemia in pregnancy: Iron, cobalamin, and folate. *Blood* 2017;129(8):940-49. doi: 10.1182/blood-2016-08-672246
20. Neogi SB, Devasenapathy N, Singh R, et al. Safety and effectiveness of intravenous iron sucrose versus standard oral iron therapy in pregnant women with moderate-to-severe anaemia in India: a multicentre, open-label, phase 3, randomised, controlled trial. *Lancet Glob Health* 2019;7(12):e1706-e16. doi: 10.1016/s2214-109x(19)30427-9 [published Online First: 2019/11/12]
21. Ortiz R, Toblli JE, Romero JD, et al. Efficacy and safety of oral iron(III) polymaltose complex versus ferrous sulfate in pregnant women with iron-deficiency anemia: a multicenter, randomized, controlled study. *J Matern Fetal Neonatal Med* 2011;24(11):1347-52. doi: 10.3109/14767058.2011.599080 [published Online First: 2011/08/24]
22. Vanobberghen F, Lweno O, Kuemmerle A, et al. Efficacy and safety of intravenous ferric carboxymaltose compared with oral iron for the treatment of iron deficiency anaemia in women after childbirth in Tanzania: a parallel-group, open-label, randomised controlled phase 3 trial. *Lancet Glob Health* 2021;9(2):e189-e98. doi: 10.1016/s2214-109x(20)30448-4 [published Online First: 2020/11/28]
23. World Bank. Prevalence of low birthweight babies (% of births) - Malawi Washington DC, USA2021 [21 August 2021]. Available from: https://data.worldbank.org/indicator/SH.STA.BRTW.ZS?locations=MW.
24. Boudová S, Divala T, Mawindo P, et al. The prevalence of malaria at first antenatal visit in Blantyre, Malawi declined following a universal bed net campaign. *Malar J* 2015;14:422. doi: 10.1186/s12936-015-0945-3 [published Online First: 2015/10/30]
25. Young MF, Oaks BM, Tandon S, et al. Maternal hemoglobin concentrations across pregnancy and maternal and child health: a systematic review and meta-analysis. *Ann N Y Acad Sci* 2019;1450(1):47-68. doi: 10.1111/nyas.14093 [published Online First: 2019/04/18]
26. Breymann C, Milman N, Mezzacasa A, et al. Ferric carboxymaltose vs. oral iron in the treatment of pregnant women with iron deficiency anemia: An international, open-label, randomized controlled trial (FER-ASAP). *Journal of Perinatal Medicine* 2017;45(4):443-53. doi: 10.1515/jpm-2016-0050

For peer review only

27. Liang K-Y, Zeger SL. Longitudinal data analysis of continuous and discrete responses for pre-post designs *Sankhyā: The Indian Journal of Statistics* 2000;Series B:134-48. doi: doi: https://www.jstor.org/stable/25053123

For peer review only

Figure heading, caption and legend

Figure 1

Figure caption and legend: *REVAMP Trial schema. V0 – visit 0; V1 – Visit 1; V2 – Visit 2; V4 – Visit 4; V7 – Visit 7; V8 – Visit 8; Abbreviations: iv – Intravenous; wks – weeks.*

For peer review only

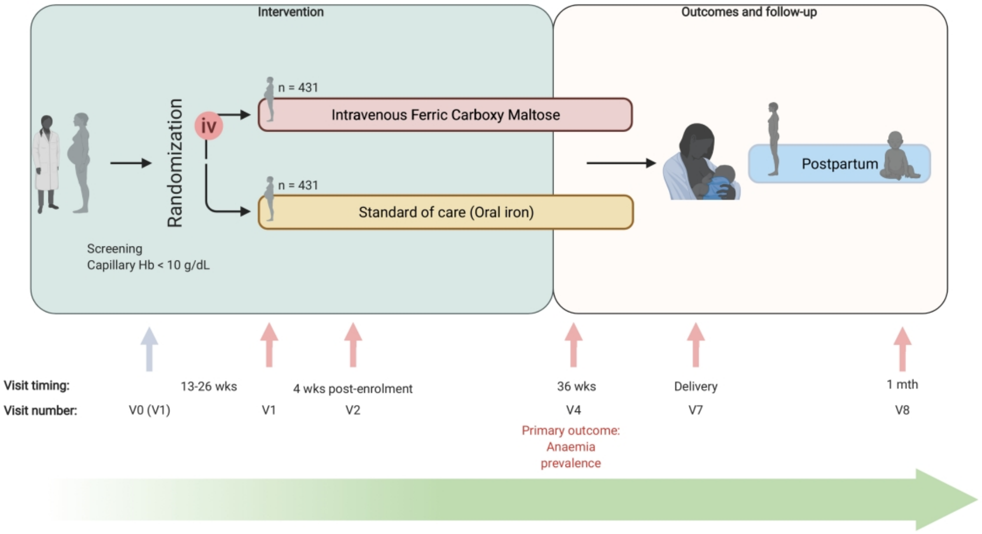

Figure 1: REVAMP Trial schema. V0 – visit 0; V1 – Visit 1; V2 – Visit 2; V4 – Visit 4; V7 – Visit 7; V8 – Visit 8; Abbreviations: iv – Intravenous; wks – weeks.

Supplementary Table 1: Primary, secondary and exploratory outcomes included in the trial registration for mothers and neonates.

	Protocol activity	Outcome type	Outcome	Timepoint
Mother	Laboratory procedures	Primary/secondary	Anaemia (Hb<11g/dL) as measured on venous blood via automated analyser	4 weeks post-intervention (for both IV iron and commencement of oral iron), week 36, at delivery, 4 weeks postpartum
		Secondary	Haemoglobin as measured on venous blood via automated analyser	4 weeks post-intervention (for both IV iron and commencement of oral iron), week 36, at delivery, 4 weeks postpartum
		Secondary	Ferritin as measured by serum ferritin	4 weeks post-intervention (for both IV iron and commencement of oral iron), week 36, at delivery, 4 weeks postpartum
		Exploratory	Iron deficiency by sTfR/Ferritin index assay	4 weeks post-intervention (for both IV iron and commencement of oral iron), week 36, at delivery, 4 weeks postpartum
		Secondary	Iron deficiency (ferritin < 15μg/L)	4 weeks post-intervention (for both IV iron and commencement of oral iron), week 36, at delivery, 4 weeks postpartum
		Exploratory	Incidence of placental malaria at delivery based on placental histologic examination	Delivery
		Exploratory	Incidence of peripheral parasitaemia by 36 weeks of gestation based on blood film microscopy	Randomisation to 36 weeks' gestation
		Exploratory	Prevalence of malaria parasitaemia based on blood film microscopy at each scheduled visit	4 weeks post-intervention (for both IV iron and commencement of oral iron), 36 weeks, at delivery, 4 weeks postpartum
		Safety	Hypophosphatemia based on biochemical measurement of serum Phosphate.	4 weeks post-intervention (for both Iv iron and commencement of oral iron), 36 weeks

For peer review only

		Safety	Inflammation (elevated C-reactive protein by serum assay)	4 weeks post-intervention (for both Iv iron and commencement of oral iron), 36 weeks' gestation
		Exploratory	Health systems costs of providing the treatments and follow-up for the intervention and comparator based on measurement of resource use and costing of relevant resources, with direct measurement of health care resource utilisation	Each planned visit that coincides with a pregnancy visit (baseline (second trimester), week 36, delivery), unplanned visits (e.g., during any episode of infection requiring management)
	Household economics	Exploratory	Direct and indirect patient costs including patient out-of-pocket costs for both health care and other costs, e.g., transport/ food, and lost income for receiving the intervention and the comparator	Each planned visit that coincides with a pregnancy visit (baseline (second trimester), week 36, delivery), unplanned visits (e.g., during any episode of infection requiring management)
		Exploratory	Fatigue measured by the Piper Fatigue Scale	4 weeks post-intervention (for both IV iron and commencement of oral iron), week 36, 4 weeks postpartum
	Maternal cognition	Exploratory	Cognitive function using digit span forward and backward test, and mental rotation tests	4 weeks post-intervention (for both IV iron and commencement of oral iron), 4 weeks postpartum
		Safety	Severe anaemia requiring blood transfusion as defined by clinical notes	From randomisation (receipt of oral or intravenous iron depending on allocation) to 4 weeks postpartum
	Adverse events	Safety	Severe medical events: shock (systolic blood pressure <90mmHg), need for blood transfusion, ICU admission, or mortality: individually and as a composite outcome, based on direct clinical observation by study staff	From randomisation (receipt of oral or intravenous iron depending on allocation) to 4 weeks postpartum

For peer review only

		Safety	Adverse events, as recorded by direct questioning of participants during administration visit. Such adverse events may include flushing, rash, allergic reactions, headache etc	Time of administration of the intervention
		Safety	Incidence of all-cause sick visits to the clinic based on visits recorded by study staff at the study clinic	Randomisation to delivery
		Exploratory	Incidence of diarrhoea sick visits to the clinic based on visits recorded by study staff at the study clinic	Randomisation to delivery
		Safety	Incidence of clinical malaria-specific sick visits to the clinic based on visits recorded by study staff at the study clinic	Randomisation to delivery
		Safety	Haemorrhage - antepartum or postpartum haemorrhage diagnosed by study clinical staff	Randomisation to 4 weeks postpartum
		Safety	Mortality	Randomisation to 1-month postpartum
		Exploratory	Shock defined by systolic blood pressure <90mmHg, as observed by study staff	Randomisation to 1-month postpartum
		Safety	Intensive care admission as observed by study staff	Recruitment to 1-month postpartum
		Safety	Need for blood transfusion, as observed by study staff	Recruitment to 1-month postpartum

For peer review only

		Safety	Delayed Adverse Events as detected by open questioning by study staff	Each scheduled visit (4 weeks post-intervention (for both IV iron and commencement of oral iron), 36 weeks, at delivery, 4 weeks postpartum
		Exploratory	Hospitalisation- any unplanned admission to hospital beyond usual postpartum discharge procedures, as observed by study staff	Following delivery
	Morbidity	Primary	Birth weight (as a continuous variable) using infant scales	Within 24 hours of birth
Neonate	Physical examination and anthropometry	Secondary	Low birth weight (birth weight <2500g) as a dichotomous variable	Within 24 hours of birth
		Exploratory	Gestational age (based on baseline ultrasound dating of pregnancy) adjusted birth weight	<24 hours following birth
		Secondary	Small for gestational age as a dichotomous variable (<10th centile)	<24 hours following birth
		Secondary	Gestation duration based on the calculated duration of gestation, using dating at baseline ultrasound examination to date of actual delivery	Delivery visit
		Secondary	Premature birth – neonate born prior to 37 completed weeks of gestation (including 36weeks and 6 days), based on gestation duration	Delivery visit
		Exploratory	Haemoglobin of venous cord blood by an automated analyser	Delivery
	Laboratory procedures	Secondary	Haemoglobin as measured on venous blood via automated analyser	1-month postpartum

For peer review only

		Exploratory	Incidence of cord blood parasitaemia at delivery based on blood film microscopy	Delivery
		Exploratory	Ferritin by serum ferritin	1 month of age
		Exploratory	Cord ferritin by serum ferritin	Delivery
		Secondary	Abortion - pregnancy loss before 28 completed weeks of gestation, as reported by the patient or based on clinical records, or as observed by study staff	<28 weeks' gestation
	Adverse events	Secondary	Stillbirth – defined as the birth of a baby showing no signs of life after 28 weeks of gestation (>28 weeks), as reported by the patient, based on clinical records, or as observed by study staff	>28 weeks' gestation
		Safety	Neonatal mortality, as observed by study staff/ clinical notes	Death of a child in the first month of life
		Safety	Neonatal intensive care admission as observed by study staff	Birth to 1-month postpartum
		Safety	Neonatal intensive care admission as observed by study staff	Birth to 1-month postpartum

For peer review only

Reporting checklist for protocol of a clinical trial.

Based on the SPIRIT guidelines.

Instructions to authors

Complete this checklist by entering the page numbers from your manuscript where readers will find each of the items listed below.

Your article may not currently address all the items on the checklist. Please modify your text to include the missing information. If you are certain that an item does not apply, please write "n/a" and provide a short explanation.

Upload your completed checklist as an extra file when you submit to a journal.

In your methods section, say that you used the SPIRITreporting guidelines, and cite them as:

Chan A-W, Tetzlaff JM, Gøtzsche PC, Altman DG, Mann H, Berlin J, Dickersin K, Hróbjartsson A, Schulz KF, Parulekar WR, Krleža-Jerić K, Laupacis A, Moher D. SPIRIT 2013 Explanation and Elaboration: Guidance for protocols of clinical trials. BMJ. 2013;346:e7586

		Reporting Item	Page Number
**Administrative information**			1
Title	#1	Descriptive title identifying the study design, population, interventions, and, if applicable, trial acronym	1
Trial registration	#2a	Trial identifier and registry name. If not yet registered, name of intended registry	2 & 4
Trial registration: data set	#2b	All items from the World Health Organization Trial Registration Data Set	4
Protocol version	#3	Date and version identifier	4
Funding	#4	Sources and types of financial, material, and other support	26
Roles and responsibilities: contributorship	#5a	Names, affiliations, and roles of protocol contributors	1 & 2
Roles and responsibilities: sponsor contact information	#5b	Name and contact information for the trial sponsor	4
Roles and responsibilities:	#5c	Role of study sponsor and funders, if any, in study design; collection, management, analysis, and interpretation of data;	4

For peer review only

sponsor and funder		writing of the report; and the decision to submit the report for publication, including whether they will have ultimate authority over any of these activities	
Roles and responsibilities: committees	#5d	Composition, roles, and responsibilities of the coordinating centre, steering committee, endpoint adjudication committee, data management team, and other individuals or groups overseeing the trial, if applicable (see Item 21a for data monitoring committee)	19
**Introduction**			
Background and rationale	#6a	Description of research question and justification for undertaking the trial, including summary of relevant studies (published and unpublished) examining benefits and harms for each intervention	7
Background and rationale: choice of comparators	#6b	Explanation for choice of comparators	20
Objectives	#7	Specific objectives or hypotheses	9,10
Trial design	#8	Description of trial design including type of trial (eg, parallel group, crossover, factorial, single group), allocation ratio, and framework (eg, superiority, equivalence, non-inferiority, exploratory)	10
**Methods: Participants, interventions, and outcomes**			
Study setting	#9	Description of study settings (eg, community clinic, academic hospital) and list of countries where data will be collected. Reference to where list of study sites can be obtained	10,11
Eligibility criteria	#10	Inclusion and exclusion criteria for participants. If applicable, eligibility criteria for study centres and individuals who will perform the interventions (eg, surgeons, psychotherapists)	11,12
Interventions: description	#11a	Interventions for each group with sufficient detail to allow replication, including how and when they will be administered	12,13
Interventions: modifications	#11b	Criteria for discontinuing or modifying allocated interventions for a given trial participant (eg, drug dose change in response to harms, participant request, or improving / worsening disease)	13
Interventions: adherance	#11c	Strategies to improve adherence to intervention protocols, and any procedures for monitoring adherence (eg, drug tablet return; laboratory tests)	13

For peer review only

Interventions: concomitant care	#11d	Relevant concomitant care and interventions that are permitted or prohibited during the trial	13
Outcomes	#12	Primary, secondary, and other outcomes, including the specific measurement variable (eg, systolic blood pressure), analysis metric (eg, change from baseline, final value, time to event), method of aggregation (eg, median, proportion), and time point for each outcome. Explanation of the clinical relevance of chosen efficacy and harm outcomes is strongly recommended	21
Participant timeline	#13	Time schedule of enrolment, interventions (including any run-ins and washouts), assessments, and visits for participants. A schematic diagram is highly recommended (see Figure)	10,18, 19,20
Sample size	#14	Estimated number of participants needed to achieve study objectives and how it was determined, including clinical and statistical assumptions supporting any sample size calculations	22, 23
Recruitment	#15	Strategies for achieving adequate participant enrolment to reach target sample size	18
**Methods: Assignment of interventions (for controlled trials)**			
Allocation: sequence generation	#16a	Method of generating the allocation sequence (eg, computer-generated random numbers), and list of any factors for stratification. To reduce predictability of a random sequence, details of any planned restriction (eg, blocking) should be provided in a separate document that is unavailable to those who enrol participants or assign interventions	13
Allocation concealment mechanism	#16b	Mechanism of implementing the allocation sequence (eg, central telephone; sequentially numbered, opaque, sealed envelopes), describing any steps to conceal the sequence until interventions are assigned	13
Allocation: implementation	#16c	Who will generate the allocation sequence, who will enrol participants, and who will assign participants to interventions	13
Blinding (masking)	#17a	Who will be blinded after assignment to interventions (eg, trial participants, care providers, outcome assessors, data analysts), and how	13,14
Blinding (masking): emergency unblinding	#17b	If blinded, circumstances under which unblinding is permissible, and procedure for revealing a participant's allocated intervention during the trial	13,14

For peer review only

Methods: Data collection, management, and analysis

Data collection plan	#18a	Plans for assessment and collection of outcome, baseline, and other trial data, including any related processes to promote data quality (eg, duplicate measurements, training of assessors) and a description of study instruments (eg, questionnaires, laboratory tests) along with their reliability and validity, if known. Reference to where data collection forms can be found, if not in the protocol	20
Data collection plan: retention	#18b	Plans to promote participant retention and complete follow-up, including list of any outcome data to be collected for participants who discontinue or deviate from intervention protocols	20,21
Data management	#19	Plans for data entry, coding, security, and storage, including any related processes to promote data quality (eg, double data entry; range checks for data values). Reference to where details of data management procedures can be found, if not in the protocol	24
Statistics: outcomes	#20a	Statistical methods for analysing primary and secondary outcomes. Reference to where other details of the statistical analysis plan can be found, if not in the protocol	23
Statistics: additional analyses	#20b	Methods for any additional analyses (eg, subgroup and adjusted analyses)	n/a: statistical analysis plan to be published separately
Statistics: analysis population and missing data	#20c	Definition of analysis population relating to protocol non-adherence (eg, as randomised analysis), and any statistical methods to handle missing data (eg, multiple imputation)	23 (SAP to be published separately)
**Methods: Monitoring**			
Data monitoring: formal committee	#21a	Composition of data monitoring committee (DMC); summary of its role and reporting structure; statement of whether it is independent from the sponsor and competing interests; and reference to where further details about its charter can be found, if not in the protocol. Alternatively, an explanation of why a DMC is not needed	19
Data monitoring: interim analysis	#21b	Description of any interim analyses and stopping guidelines, including who will have access to these interim results and make the final decision to terminate the trial	23,24
Harms	#22	Plans for collecting, assessing, reporting, and managing solicited	21,22

For peer review only

		and spontaneously reported adverse events and other unintended effects of trial interventions or trial conduct	
Auditing	#23	Frequency and procedures for auditing trial conduct, if any, and whether the process will be independent from investigators and the sponsor	19
**Ethics and dissemination**			
Research ethics approval	#24	Plans for seeking research ethics committee / institutional review board (REC / IRB) approval	4
Protocol amendments	#25	Plans for communicating important protocol modifications (eg, changes to eligibility criteria, outcomes, analyses) to relevant parties (eg, investigators, REC / IRBs, trial participants, trial registries, journals, regulators)	4
Consent or assent	#26a	Who will obtain informed consent or assent from potential trial participants or authorised surrogates, and how (see Item 32)	See supplementary material 1
Consent or assent: ancillary studies	#26b	Additional consent provisions for collection and use of participant data and biological specimens in ancillary studies, if applicable	See supp. Material 1
Confidentiality	#27	How personal information about potential and enrolled participants will be collected, shared, and maintained in order to protect confidentiality before, during, and after the trial	23,24
Declaration of interests	#28	Financial and other competing interests for principal investigators for the overall trial and each study site	25
Data access	#29	Statement of who will have access to the final trial dataset, and disclosure of contractual agreements that limit such access for investigators	23,24
Ancillary and post trial care	#30	Provisions, if any, for ancillary and post-trial care, and for compensation to those who suffer harm from trial participation	21
Dissemination policy: trial results	#31a	Plans for investigators and sponsor to communicate trial results to participants, healthcare professionals, the public, and other relevant groups (eg, via publication, reporting in results databases, or other data sharing arrangements), including any publication restrictions	8,24
Dissemination policy: authorship	#31b	Authorship eligibility guidelines and any intended use of professional writers	24

For peer review only

Dissemination policy: reproducible research	#31c	Plans, if any, for granting public access to the full protocol, participant-level dataset, and statistical code	n/a; statistical analyses plan to be published separately
**Appendices**			
Informed consent materials	#32	Model consent form and other related documentation given to participants and authorised surrogates	Supplementary material 1
Biological specimens	#33	Plans for collection, laboratory evaluation, and storage of biological specimens for genetic or molecular analysis in the current trial and for future use in ancillary studies, if applicable	14,15, 16

None The SPIRIT Explanation and Elaboration paper is distributed under the terms of the Creative Commons Attribution License CC-BY-NC. This checklist can be completed online using https://www.goodreports.org/, a tool made by the EQUATOR Network in collaboration with Penelope.ai

For peer review only
